# Persistent enrichment of multidrug-resistant *Klebsiella* in oral and nasal communities during long-term starvation

**DOI:** 10.1186/s40168-024-01854-5

**Published:** 2024-07-20

**Authors:** Jett Liu, Nell Spencer, Daniel R. Utter, Alex S. Grossman, Lei Lei, Nídia Castro dos Santos, Wenyuan Shi, Jonathon L. Baker, Hatice Hasturk, Xuesong He, Batbileg Bor

**Affiliations:** 1 Department of Microbiology, ADA Forsyth Institute, Cambridge, MA 02142 USA; 2 Division of Geological and Planetary Sciences, California Institute of Technology, Pasadena, CA 91125 USA; 3 State Key Laboratory of Oral Diseases & National Clinical Research Center for Oral Diseases &, Department of Preventive Dentistry, West China Hospital of Stomatology, Sichuan University, Chengdu, 610041 China; 4 Dental Research Division, Guarulhos University, Guarulhos, São Paulo Brazil; 5 Albert Einstein School of Dental Medicine, Albert Einstein Israelite Hospital, São Paulo, SP Brazil; 6 Department of Oral Rehabilitation & Biosciences, Oregon Health & Science University, Portland, OR 97239 USA; 7 Center for Clinical and Translational Research, ADA Forsyth Institute, Cambridge, MA 02142 USA

**Keywords:** *Klebsiella*, Oral microbiome, Nasal microbiome, Nosocomial infection, Opportunistic pathogen, Microbiome ecology, Multidrug resistance, Commensal bacteria

## Abstract

**Background:**

The human oral and nasal cavities can act as reservoirs for opportunistic pathogens capable of causing acute infection. These microbes asymptomatically colonize the human oral and nasal cavities which facilitates transmission within human populations via the environment, and they routinely possess clinically significant antibiotic resistance genes. Among these opportunistic pathogens, the *Klebsiella* genus stands out as a notable example, with its members frequently linked to nosocomial infections and multidrug resistance. As with many colonizing opportunistic pathogens, the essential transmission factors influencing the spread of *Klebsiella* species among both healthy and diseased individuals remain unclear.

**Results:**

Here, we explored a possible explanation by investigating the ability of oral and nasal *Klebsiella* species to outcompete their native microbial community members under *in vitro* starvation conditions, which could be analogous to external hospital environments or the microenvironment of mechanical ventilators. When *K. pneumoniae* and *K. aerogenes* were present within a healthy human oral or nasal sample, the bacterial community composition shifted dramatically under starvation conditions and typically became enriched in *Klebsiella* species. Furthermore, introducing *K. pneumoniae* exogenously into a native microbial community lacking *K. pneumoniae*, even at low inoculum, led to repeated enrichment under starvation. Precise monitoring of *K. pneumoniae* within these communities undergoing starvation indicated rapid initial growth and prolonged viability compared to other members of the microbiome. *K. pneumoniae* strains isolated from healthy individuals’ oral and nasal cavities also exhibited resistance to multiple classes of antibiotics and were genetically similar to clinical and gut isolates. In addition, we found that in the absence of *Klebsiella* species, other understudied opportunistic pathogens, such as *Peptostreptococcus*, increased in relative abundance under starvation conditions.

**Conclusions:**

Our findings establish an environmental and microbiome community circumstance that allows for the enrichment of *Klebsiella* species and other opportunistic pathogens. *Klebsiella*’s enrichment may hinge on its ability to quickly outgrow other members of the microbiome. The ability to outcompete other commensal bacteria and to persist under harsh environmental conditions could be an important factor that contributes to enhanced transmission in both commensal and pathogenic contexts.

Video Abstract

**Supplementary Information:**

The online version contains supplementary material available at 10.1186/s40168-024-01854-5.

## Background

Microbes that are typically of low abundance and harmless in their normal niche, but potentially dangerous under specific circumstances are termed colonizing opportunistic pathogens (COPs) [1]. Translocation of human oral bacteria to other body sites has been associated with noncommunicable, chronic systemic illnesses including diabetes, cancer, and Alzheimer’s disease [2–5]. The oral microbiota also contains potential opportunistic pathogens that can cause more acute diseases [6]. These oral COPs include pathogens such as *Streptococcus pyogenes*, *Streptococcus pneumoniae*, and *Haemophilus influenzae*, which are part of the normal oral community but can cause numerous diseases, including but not limited to streptococcal pharyngitis, pneumonia, sepsis, and/or meningitis [7]. Crucially, oral microbes and COPs can asymptomatically colonize and transmit between people, complicating their disease etiology [8]. Characterizing how oral COPs colonize, transmit, and transition from colonization to infection is crucial, given that human oral fluids can contaminate surfaces and be ingested in the body [9–12].

*Klebsiella* species are a prominent example of an oral COP. Typically, oral *Klebsiella* species exhibit a low prevalence and abundance in populations studied to date [13–15], having an increased association with periodontal pockets [14, 16, 17]. Despite their relatively low prevalence in the oral microbiome, whenever they are found, *Klebsiella* seem to be a consistent colonizer and member of the community [15]. Oral fluids are a major source of contamination in the healthcare setting, particularly in sinks and drains [12, 18], and previous studies have demonstrated that *Klebsiella* species can persist and transmit particularly well in nosocomial environments [19, 20]. Therefore, a small number of individuals carrying oral *Klebsiella* species could potentially act as superspreaders, seeding colonization and infection within a larger population and among at-risk persons.

Although routinely carried asymptomatically, *Klebsiella pneumoniae* is one of the six ESKAPE pathogens that can be highly virulent and are routinely resistant to multiple antibiotics [21, 22]. Additionally, other species within *Klebsiella*, such as *K. variicola*, *K. aerogenes*, and *K. oxytoca,* show similar drug-resistant and virulent phenotypes [21]. As a result, *Klebsiella* species are a major cause of life-threatening hospital-acquired infections in at-risk immunocompromised and critically ill patients [23]. They are also associated with various gastrointestinal infections, which can lead to gastroenteritis, colitis, and other chronic diseases [24]. The origin of *Klebsiella* infection has been linked to environmental samples (including soils, plants, and animals), human body sites (including the gut and skin microbiomes), and hospital environments (including contaminated sinks and drains) [13, 19–21, 25, 26]. Despite the strong linkages between *Klebsiella* and nosocomial infections, the role of the human oral cavity as a reservoir for *Klebsiella* species associated with nosocomial and gut infections has been largely overlooked. Most research on oral *Klebsiella* as an opportunistic pathogen was conducted in mouse studies and has demonstrated that mouse or human oral *Klebsiella* species can migrate to the mouse gut and cause various inflammation and colitis [27, 28].

Illustrating their toughness and ability to survive in various stress conditions, numerous nosocomial pathogens, including *Klebsiella*, have been shown to persist on various abiotic surfaces [29, 30]. These bacteria possess various mechanisms to withstand environmental stress, enabling them to survive challenging conditions for varying durations. While these adaptations may have evolved in response to environmental stress, many environmental stress responses are often useful for pathogenic niches, including desiccation resistance [31], chemical competition [32], and persistence states [33]. Our previous study examined the oral microbiome community under starvation conditions akin to those of an expelled oral droplet persisting on a surface [34]. We found that members of the Enterobacteriaceae family, and particularly *Klebsiella* species, outlasted other bacterial community members to emerge as the only surviving taxa. This finding was assessed using metagenomics, metatranscriptomics, and traditional growth methods; confirming that, among the studied samples, Enterobacteriaceae were the majority of the transcriptionally active bacteria after 100 days of starvation, and the only taxa that could be recovered by plating.

The previous study, however, was limited by a small sample size and did not examine the ubiquity of the Enterobacteriaceae survival phenomenon. In this study, we further assessed the capability of *Klebsiella* species to persist under starvation conditions by including additional clinical samples from the oral cavity. We also examined nares samples given that nares fluids can contaminate hospital surfaces. We observed that when *Klebsiella* species were present within a community, they consistently enriched in starved communities, especially *Klebsiella pneumoniae*, which not only endured starvation but also proliferated rapidly to establish dominance within the first 24 h of starvation. Furthermore, all cultured strains of *Klebsiella pneumoniae* isolated from healthy human oral and nasal cavities exhibited multidrug resistance. These strains were found to be phylogenetically intermingled with previously sequenced clinically relevant and healthy gut isolates. In the absence of *Klebsiella* species, other bacteria such as *Peptostreptococcus* were enriched in starved communities. These findings collectively suggest that oral COPs, such as *Klebsiella* species, have the capability to persist longer than other members of oral and nasal microbial communities under starvation conditions. This enduring trait might have the potential to influence the colonization-to-infection process, especially in areas where the opportunistic pathogen has to go through starvation or other stress environments.

## Results

### Starvation of oral and nasal polymicrobial communities in vitro reveals enriched genera

**Fig. 1:**
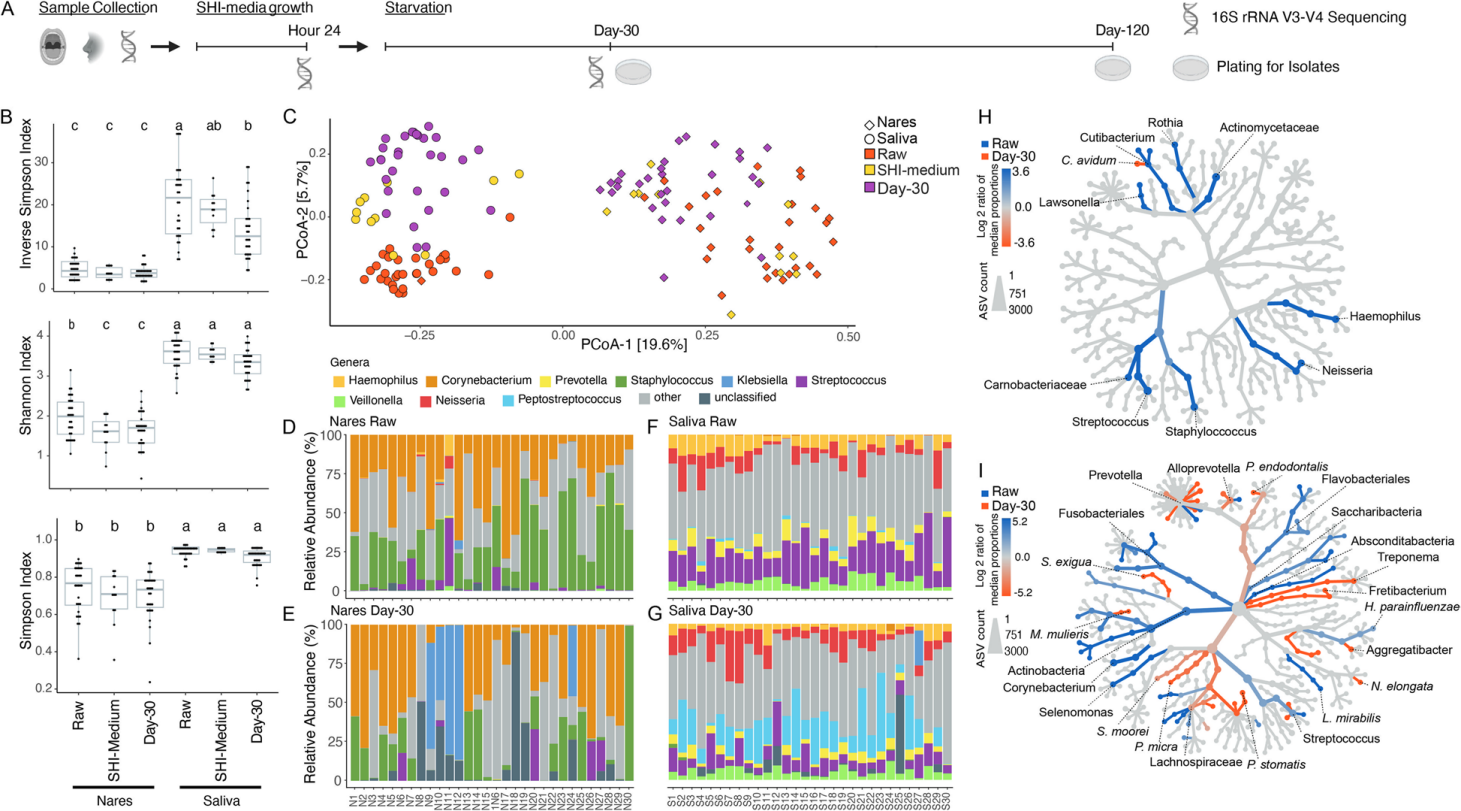
Community and relative abundance analysis of sequenced samples. **A** Timeline detailing sample collection, SHI-medium growth, and starvation procedures. Sequencing and plating times are indicated. **B** Alpha diversity analysis of sequenced samples grouped by culture condition and body site. Alpha diversity was calculated using ASV abundances. **C** Principal coordinates analysis (PCoA) plot produced using the Bray-Curtis distance function and ASV abundances. Body site and culture condition are indicated by color. **D**–**G** Stacking bar charts detailing the relative abundance of genera identified within sequenced samples. **H** Comparative analysis of bacteria gained or lost between nares raw communities and day-30 communities. The color indicates lost or gained taxon groups on a log_2_ median proportion scale. The node size indicates normalized ASV counts. Only taxon levels with a Wilcoxon rank-sum *p-*value ≤ 0.05 are colored. **I** Comparative analysis of bacteria gained or lost between saliva raw communities and day-30 communities. The color indicates lost or gained taxon groups on a log_2_ median proportion scale. The node size indicates normalized ASV counts. Only taxon levels with a Wilcoxon rank-sum *p*-value ≤ 0.05 are colored

Thirty healthy human volunteers were recruited for the sampling of two body sites, saliva (S1–S30) and nares (N1–N30), for a total of sixty samples. These initial oral and nasal communities (hereafter referred to as the raw communities) were inoculated into SHI-medium, and the resultant communities were incubated for 24 h (hereafter referred to as the SHI-medium communities). SHI-medium has been extensively used to expand the biomass of oral microbial communities with minimal impact on biodiversity [35]. Subsequently, the SHI-medium communities were resuspended and aliquoted in PBS and starved under aerobic conditions for both 30 days (hereafter referred to as the day-30 communities) and 120 days (hereafter referred to as the day-120 communities) (Fig. 1A).

To initially assess how starvation conditions alter the composition of oral and nasal communities, we performed 16S rRNA gene sequencing of the V3–V4 regions on all raw communities, several SHI-medium communities, and all day-30 communities. Using identified ASVs, we analyzed the alpha (intrasample) diversity of the different communities using the Shannon, Simpson, and Inverse Simpson indices. Broadly, nares samples were less diverse than saliva samples (Fig. 1B), an expected finding that has been reported in other studies [36]. Across both nares and saliva samples, we observed a slight decrease in alpha diversity when comparing the raw communities to the SHI-medium communities (Fig. 1B). Further, both nares and saliva day-30 communities tended to exhibit slightly lower alpha diversity when compared to the SHI-medium communities (Fig. 1B). Altogether, the alpha diversity metrics generally indicated that both nares and saliva communities tended to decrease in alpha diversity when subjected to starvation conditions. Beta diversity (intercommunity) plots based on identified ASVs displayed a clear separation between raw nares and saliva communities (PERMANOVA, *p* ≤ 0.001), an expected result given the known difference in bacterial community composition across body sites (Fig. 1C). Similarly, samples from the same body site are typically clustered according to culture condition (Fig. 1C), an observation particularly pronounced among the nares samples. In general, SHI-medium communities had a less pronounced shift away from the raw communities when compared to day-30 communities, which clearly clustered apart from their raw community counterparts.

We next analyzed the genera-level relative abundance data, which revealed several interesting trends. Although the nares samples did not appear to display a consistent increase in a particular genus when comparing the raw communities to the day-30 communities, it was striking to observe that five of the thirty day-30 communities (N9, N10, N11, N12, and N24) contained a large relative abundance—greater than 45%—of *Klebsiella* (Fig. 1D,E; Additional file 1: Tables S1, S2, and S3). This large increase in *Klebsiella* abundance was even more pronounced when considering that only six raw nares communities (samples N9, N10, N11, N12, N17, and N24) contained detectable levels of *Klebsiella,* with a mean relative abundance of ~1.28% (0.1–5.7% range). In contrast to the nares samples, saliva communities displayed more consistent changes in genera-level relative abundances. In particular, we noted that compared to raw saliva communities, day-30 communities decreased in *Streptococcus* relative abundance and increased in *Peptostreptococcus* relative abundance (Fig. 1F,G, Additional file 1: Tables S1, S2, and S3). We observed only one saliva sample (S27) had detectable *Klebsiella* in the raw communities, and its abundance increased to ~22.5% of the day-30 community (Fig. 1G). Interestingly, we did not detect *Klebsiella* in both the oral and nasal cavities of the same individual in any of the samples.

Global comparative analysis of community composition confirmed these initial observations. Nares samples displayed few consistent statistically significant changes when subjected to starvation (Wilcox rank sum ≤ 0.05) (Fig. 1H, Additional file 1: Table S4). In contrast, saliva samples were much more consistent in statistically significant community composition changes between raw and starvation conditions (Fig. 1I; Additional file 1: Table S5).

### Opportunistic pathogens enriched in starved communities

**Fig. 2:**
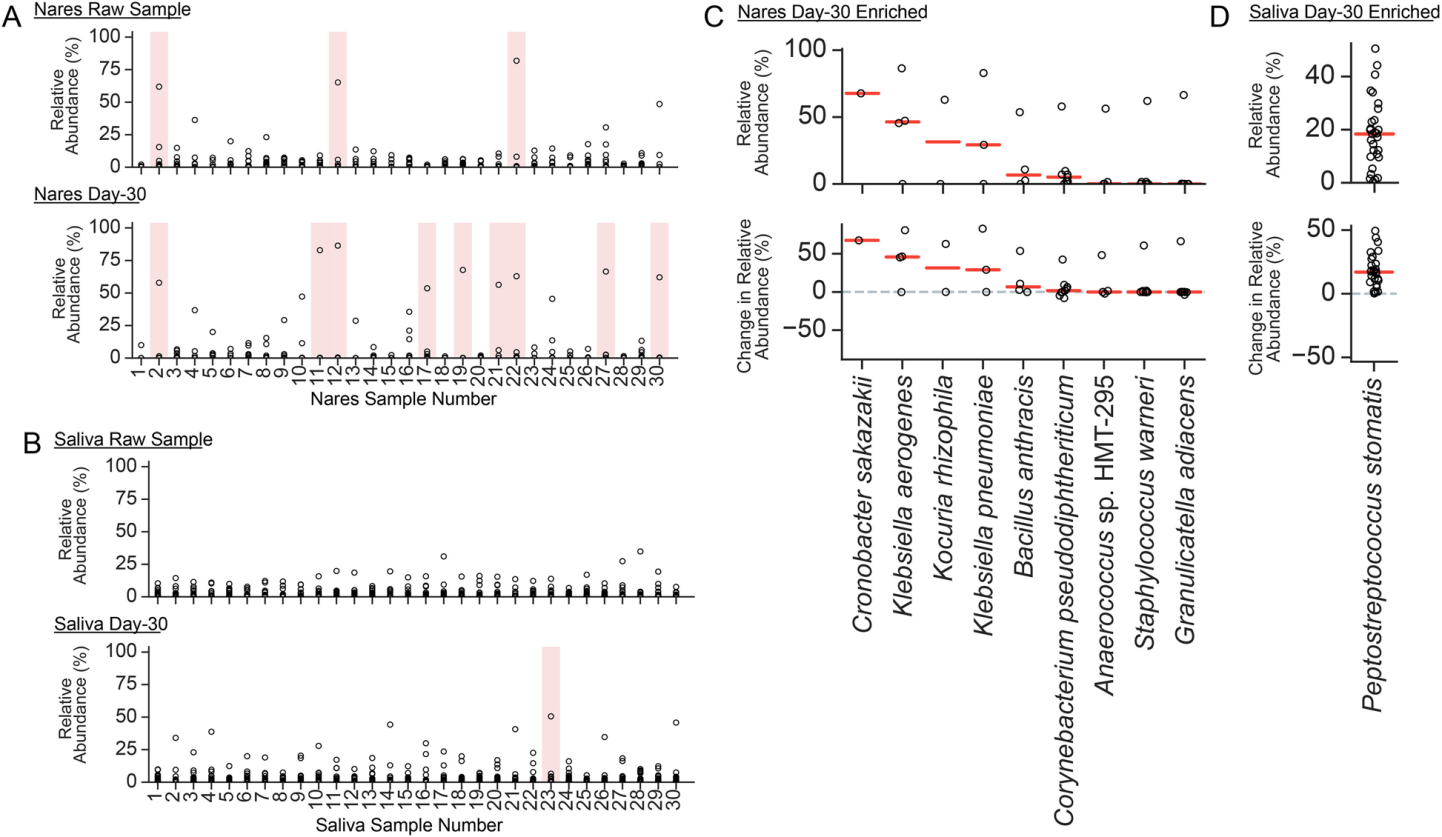
Species-level analysis on enriched community members under stressed conditions. **A** Nares raw community and day-30 community species-based relative abundance analysis. Each point represents a single species. Samples (columns) with at least one species ≥ 50% relative abundance are shaded pink. **B** Saliva raw community and day-30 community species-based relative abundance analysis. Each point represents a single species. Samples (columns) with at least one species ≥ 50% relative abundance are shaded pink. **C** Relative abundance analysis of Nares species with ≥ 50% relative abundance in at least one day-30 community (shaded pink from panel **A**). The top panel displays day-30 relative abundances. The bottom panel displays the change in relative abundance from raw communities to day-30 communities. **D** Relative abundance analysis of saliva species with ≥ 50% relative abundance in at least one day-30 community (shaded pink from panel **B**). The top panel displays day-30 relative abundances. The bottom panel displays the change in relative abundance from raw communities to day-30 communities

Particularly intrigued by the dominance of *Klebsiella* in several of the nares and one saliva day-30 communities, we sought to unbiasedly assess if specific species were enriched in the starved communities (Fig. 2A,B). At the species level, three nares raw communities contained a single species with above 50% relative abundance (Fig. 2A, Additional file 1: Table S6). These initially dominant species were *Dolosigranulum pigrum* (N2 and N22) and *Corynebacterium tuberculostearicum* (N12). Strikingly, after starvation, nine nares day-30 communities contained a single species with over 50% relative abundance (Fig. 2A, Additional file 1: Tables S7 and S8). These nine dominant species were *Klebsiella pneumoniae*, *Klebsiella aerogenes*, *Kocuria rhizophila*, *Cronobacter sakazakii*, *Bacillus anthracis*, *Corynebacterium pseudodiphtheriticum*, *Anaerococcus* sp. HMT-295, *Staphylococcus warneri*, and *Granulicatella adiacens* (Fig. 2C). Notably, many of these species, including *Klebsiella*, are known pathogens that have the ability to survive challenging thermal, desiccation, and pH stress environments [37–40]. Additionally, some of these species are not well-known nasal commensals but have been detected sporadically [41]. This dominance of a particular species within a day-30 community was typically a single occurrence per taxa—7 of the 9 dominant species had a relative abundance above 50% in only a single day-30 community (*K. rhizophila*, *C. skazakii*, *B. anthracis*, *C. pseudodiphtheriticum*, *A.* sp. HMT-295, *S. warneri*, and *G. adiacens*). Both *Klebsiella* species, *K. pneumoniae*, and *K. aerogenes*, however, were present in multiple samples and displayed a consistent increase in relative abundance after starvation (Fig. 2C).

In contrast to the nares samples, the number of saliva samples with a dominant species above 50% relative abundance increased from zero in raw communities to one in day-30 community (Fig. 2B, Additional file 1: Tables S6, S7, and S8). This sample, day-30 S23, had a *P. stomatis* relative abundance greater than 50%. Additionally, across all samples, *P. stomatis* displayed a mean increase in relative abundance of 17.8% from raw to day-30 conditions (Fig. 2D).

### Solid media growth of the starved communities largely support 16S rRNA sequencing results and were enriched in *Klebsiella* species

**Fig. 3:**
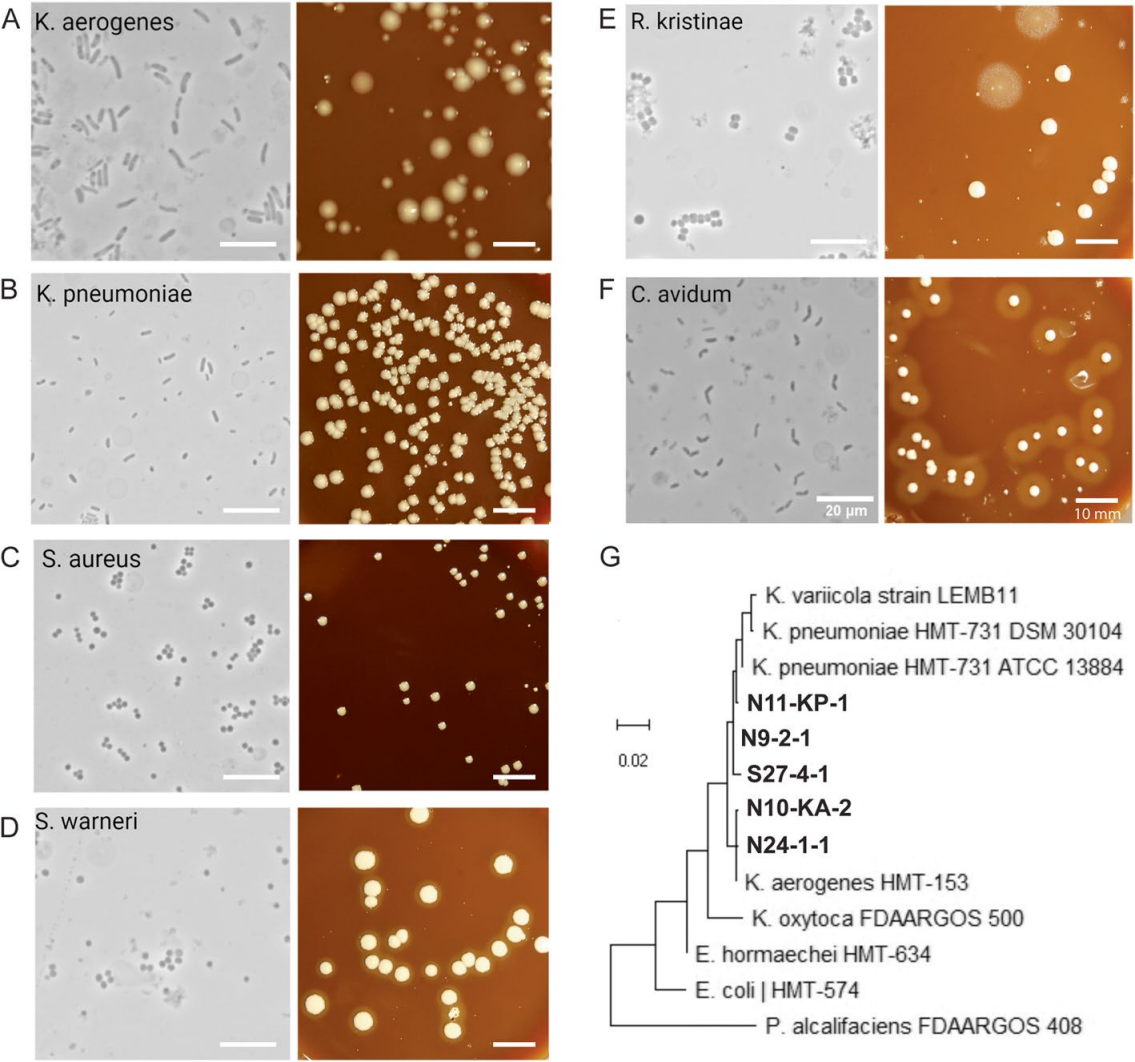
Growth of day-30 and day-120 communities on solid media. **A**–**F** Phase-contrast and colony images of isolated bacterial strains from the starved day-30 and day-120 communities. These are representative images of a larger cohort, which is summarized in Additional file 1: Table S9. **G** Neighbor-joining tree for partial 16S rRNA sequences using MEGAX. Isolated bacterial strains from this study are designated by their nares (N) and saliva (S) subject numbers in bold and combined with representative reference strains from eHOMD. Only unique *Klebsiella* species from different saliva and nares samples were included. All scale bars are 20 μm for phase-contrast images and 10 mm for colony images

16S rRNA gene sequencing detects both live and dead bacteria. To survey viable bacteria, we cultured communities from raw, day-30, and day-120 on blood BHI agar plates microaerophilically. Before starvation, raw communities exhibited a wide range of colony diversity; they varied broadly in size, color, and texture (Additional file 2: Figure S1A). However, after 30 and 120 days of starvation, colony diversity decreased drastically, and we mostly observed uniform mucoid colonies (Fig. 3, Additional file 2: Fig. S1B-C). To identify the day-30 and day-120 colonies, we performed partial 16S amplification and Sanger sequencing on 91 colonies. In samples that had enriched *Klebsiella* species by 16S rRNA profiling, the vast majority of the colonies from the starved communities were *Klebsiella* species (Fig. 3A, B, Additional file 1: Table S9). Specifically, two dominant *Klebsiella* species from independent samples emerged in our cultures, *K. pneumoniae* (samples N9, N11, and S27) and *K. aerogenes* (samples N10 and N24). When placed in a 16S rRNA phylogenetic tree, the sequenced *Klebsiella* species formed two main clades accordingly (Fig. 3G). Moreover, we identified and isolated multiple *Staphylococcus* species in both nares and saliva samples, *Rothia* from saliva samples, and *Cutibacterium* from nares samples (Fig. 3C–F, Additional file 1: Table S9). We did not observe the growth of *Peptostreptococcus*, generally considered a strict anaerobe, suggesting that the microaerophilic growth conditions did not support their growth [42]. Despite the limitations of blood BHI agar plates and microaerophilic conditions to support the growth of many saliva and nares bacteria, *Klebsiella* species enrichment under starvation conditions, compared to raw communities, was very dramatic, suggesting that when present, even in small initial abundance, *Klebsiella* can take over the community and stay viable after starvation. In starvation cultures that did not have *Klebsiella* species, we observed mixed colonies that have heterogeneous morphologies.

### *Klebsiella pneumoniae* enriches in nares and saliva communities with high efficiency under starvation conditions

**Fig. 4:**
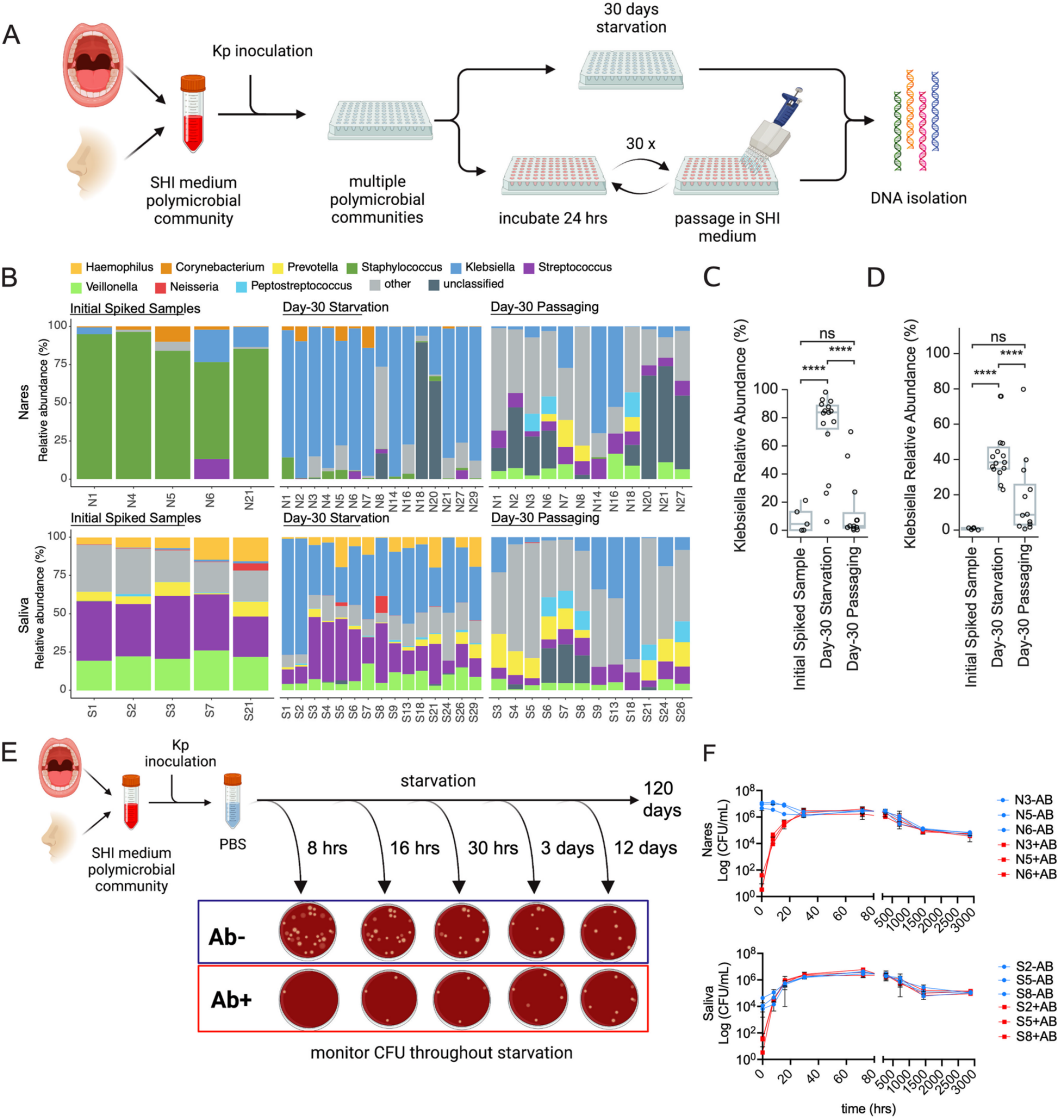
Artificial spiking of oral and nasal polymicrobial communities with *K. pneumoniae* results in consistent takeover. **A** Cartoon depiction of *K. pneumoniae* spiking experiment. Fifteen saliva and 15 nares samples were spiked with low abundance of *K. pneumoniae* strains and incubated by two different methods simultaneously: 30 days of starvation or 30 days of nutrition-rich passaging in SHI media. **B** 16S relative abundance data of the spiking experiment after 30 days of starvation. Five samples of the initial spiked but not incubated community were shown as a control. **C**, **D** Relative abundance of only *K. pneumoniae* plotted from panel **B** for all three groups. Pairwise statistical comparisons were performed using Student’s *t* test. “****” indicates a *p* value less than or equal to 10^−4^. “ns” denotes a *p* value greater than 0.05. **E** Cartoon depiction of spiking experiment with determining viable colony-forming units (CFU) of *K. pneumoniae* and other community members at each time point with (+AB) or without (−AB) antibiotic challenge. **F** CFUs were plotted against the time of plating. Both CFU on the BHI blood plate (blue, −AB) and antibiotic cocktail plate (red, +AB) are shown for three different SHI media communities of saliva and nares samples. We expect only antibiotic-resistant *K. pneumoniae* will grow on +AB platess

Considering that *Klebsiella* species were infrequently found in saliva and nares communities (1/30 samples and 5/30 samples), we aimed to strengthen the significance of our findings by conducting *in vitro* experiments. We randomly selected 15 saliva and 15 nares SHI-medium communities and grew them under microaerophilic conditions to mimic their native low-oxygen environment. These communities lacked any presence of *Klebsiella* species and we spiked these samples with 0.1% relative abundance of *K. pneumoniae* strain N9-2-1 (Fig. 4A). We picked N9-2-1 since, out of the three well-characterized *K. pneumoniae* strains in our study*,* it was the only strain that isolated from the day-120 starvation (Additional file 1: Table S9). We reasoned that this strain would have a more selective advantage compared to day-30 strains and would show a clearer phenotype. The estimate of 0.1% was first determined from the 16S rRNA profiling of the raw sequence in Fig. 1, and then, optical density measurement and colony-forming units were used to estimate the final spiking (see the “ Methods” section). Post-spiking but before starvation, the *K. pneumoniae* relative abundance by 16S rRNA sequencing across the communities ranged from 0.04 to 21.0% (mean 4.3% relative abundance) (Fig. 4B–D, Additional file 1: Table S10).

Subsequently, we subjected each spiked community to two conditions: one aliquot was starved for 30 days, while another was passaged in fresh SHI-medium at a 1:5 dilution every 24 h for 30 days. The starvation setup and processing were conducted mostly in room air to mimic external environmental contamination while passaging was conducted under microaerophilic conditions to mimic oral and nasal cavities (see the “ Methods” section). Afterward, we analyzed the resulting communities by 16S rRNA gene sequencing. Strikingly, similar to the above starvation experiments, exogenously added *K. pneumoniae* expanded in almost all starved saliva and nares communities. Spiked and starved saliva communities contained a mean *K. pneumoniae* relative abundance of 42.7% (range 22.8–75.8%), and spiked nares communities contained a mean *K. pneumoniae* relative abundance of 72.2% (range 6.2–98.2%) (Fig. 4B–D, Additional file 1: Table S11). In contrast, the spiked communities that were passaged daily using fresh SHI-medium did not experience *K. pneumoniae* outgrowth to the same extent. Passaged spiked saliva communities contained a mean *K. pneumoniae* relative abundance of 18.8% (range 0.66–79.8%) and passaged spiked nares communities contained a mean *K. pneumoniae* relative abundance of 14.7% (range 0.25–70.0%) (Fig. 4B–D, Additional file 1: Table S12). We want to note that the SHI-medium passaging cohort did not experience a completely ideal nutrition-rich condition, as these cultures went through lag, exponential, and brief stationary phases during each passage. Nevertheless, despite these limitations, the results from both conditions clearly indicated that *K. pneumoniae* does not consistently increase in saliva or nares communities under nutrition-rich conditions. These 16S data were also supported by denaturing gradient gel electrophoresis (DGGE) band intensity analysis of communities N6, N21, S3, and S27 (Additional file 2: Fig. S2A).

Having obtained *K. pneumoniae* isolates from the healthy human oral cavity, we capitalized on the opportunity to experimentally characterize the antibiotic resistance of our isolated *K. pneumoniae* strains*.* Initially, we evaluated the antibiotic and antibiotic cocktail resistance of *K. pneumoniae* strains from three healthy individuals (N9-2-1, N11-KP-1, S27-4-1) (Additional file 1: Table S9). We compared these *K. pneumoniae* strains to the resistance of three SHI-media saliva and nares communities that did not contain any *Klebsiella* species. As expected, the *K. pneumoniae* strains demonstrated resistance to ampicillin, vancomycin, and the antibiotic cocktail comprising vancomycin, trimethoprim, cefsulodin, and amphotericin B (Additional file 2: Fig. S2B). Additionally, two strains (N9-2-1 and N11-KP-1) displayed partial resistance to nalidixic acid, and a single strain (N9-2-1) displayed resistance to erythromycin. Surprisingly, the three nares (N3, N5, N6) and three saliva (S2, S5, S8) SHI-medium communities were largely susceptible to all antibiotics that we tested.

Leveraging the antibiotic resistance of these strains and the antibiotic susceptibility of the nares and saliva communities, we conducted another spiking experiment to further confirm that *K. pneumoniae* was increasing in starvation communities when present. We again inoculated saliva (S2, S5, S8) and nares (N3, N5, N6) communities with *K. pneumoniae* N9-2-1 before subjecting the spiked communities to up to 120 days of starvation (Fig. 4E). At several time points during the 120-day starvation, we plated each spiked community onto two types of blood agar plates: one that contained no antibiotics and one that contained the four-antibiotic cocktail of vancomycin, trimethoprim, cefsulodin, and amphotericin B. We determined the communities’ colony-forming units (CFU) on each plate, which allowed us to assess the approximate relative abundance of viable S9-2-1 in the communities during starvation. At the very early stages of starvation, we observed the presence of diverse colony morphology types distinct from the typical mucoid *K. pneumoniae* colonies on the antibiotic-negative plates (Additional file 2: Fig. S3A). Throughout the starvation period, *K. pneumoniae* initially displayed a low CFU count but quickly grew to take over the communities, within ~30 h and ~8 h for nares and saliva, respectively (Fig. 4F). It is worth noting that in saliva communities, *K. pneumoniae* grew to an even higher CFU count than the starting CFU count of the community, suggesting that either *K. pneumoniae* has a remarkable capability to take over in starvation, or a large portion of saliva community members such as *Peptostreptococcus* could not be cultured on blood BHI agar plates (Fig. 4F, Additional file 2: Fig. S3A). *K. pneumoniae* also managed to maintain a high viability throughout the entire starvation period. Starved day-60 and day-120 communities still contained ~10^6^ CFUs, and the majority of them were mucoid colonies. Prior research has demonstrated that certain bacteria can grow on nutrients released by deceased bacteria [43]. To explore this hypothesis, we replicated the starvation experiment, but supplemented the culture with either heat-killed *K. pneumoniae* or a heat-killed saliva S2 community. As anticipated, *K. pneumoniae* exhibited comparable growth levels when supplemented with the heat-killed or live S2 communities, indicating *K. pneumoniae’s* ability to utilize nutrients from dead bacteria (Additional file 2: Fig. S3B). As a control*, K. pneumoniae* did not demonstrate growth when cultured alone in a buffer without any supplementation of dead or live bacterial communities (Additional file 2: Fig. S3C)*.* In these experiments, we subjected another *K. pneumoniae* strain, S27-4-1, to starvation to demonstrate that our findings were repeatable with multiple *K. pneumoniae* strains. These results reaffirm our 16S sequencing analysis and indicate that the increased relative abundance of *K. pneumoniae* at the conclusion of the starvation period is attributed to both the early growth and prolonged survival capacity of these bacteria.

### Pangenome analysis of *K. pneumoniae* isolates reveal conserved pathogenic and antibiotic resistance genes compared to clinical isolates

**Fig. 5:**
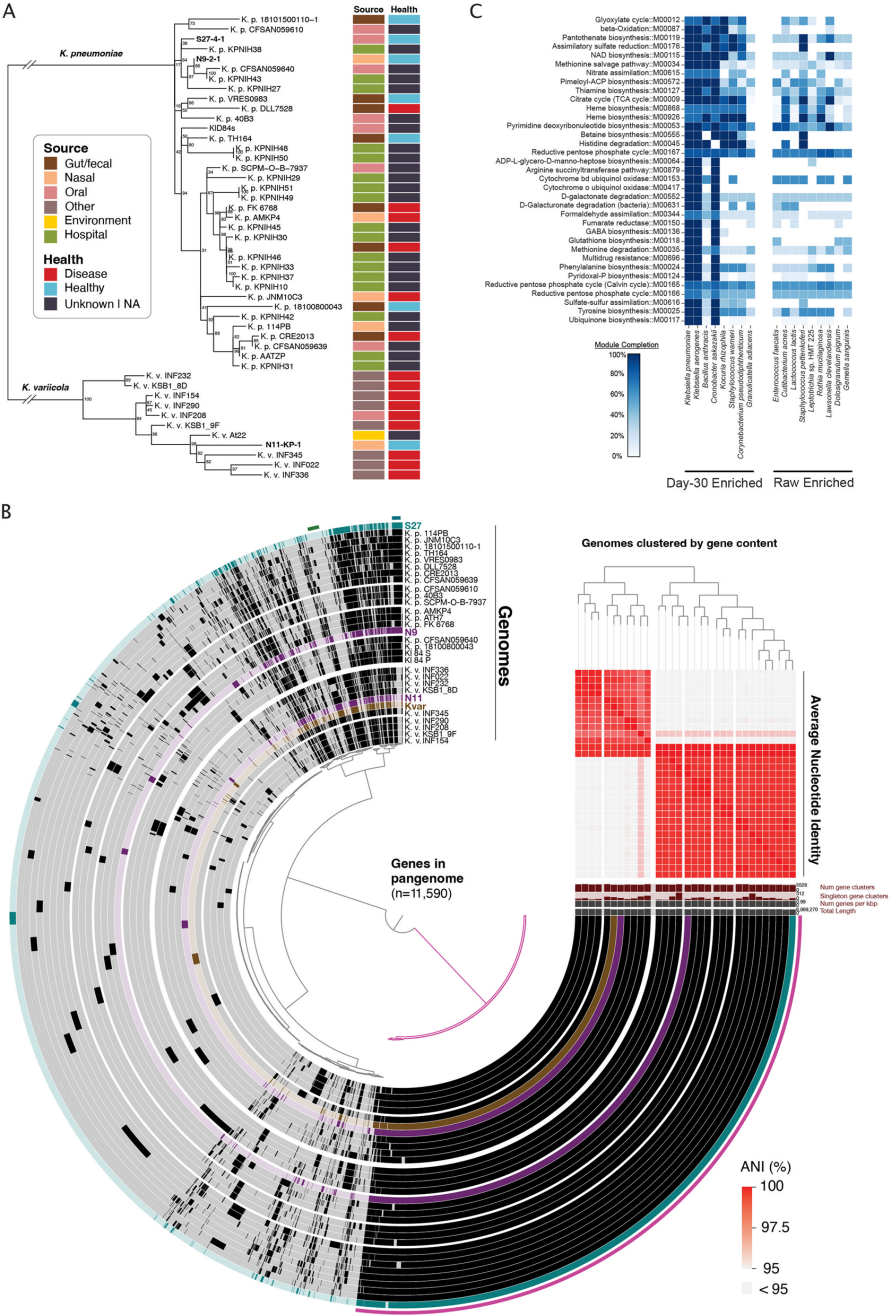
Genomic analysis of *K. pneumoniae* and *K. variicola*. **A** Maximum-likelihood tree of *Klebsiella* isolates from this study along with public reference genomes. Node labels represent bootstraps. Color boxes next to tips show the strain’s isolation source (first column) and the health status of the host (second column). **B** Pangenome of select *K. pneumoniae* and *K. variicola* isolates. Each track represents a different genome, and each unit along the track (i.e., inner dendrogram) represents a different gene homolog. Reference genomes are colored black, N9-2-1 and N11-KP-1 are purple, S27-4-1 is teal, and At22 (ant symbiont) is brown*.* Genomes are arranged by the presence/absence of genes (upper right dendrogram). The heatmap shows average nucleotide identity (ANI) between strains, clipped 95–100%. **C** Whole genome functional enrichment analysis of starvation-enriched species compared to starvation-depleted species. KEGG modules enriched among starvation-enriched genomes with a *p-*value of < 0.05 are displayed. Color intensity signifies module completeness

Among our cultured *K. pneumoniae* isolates, we performed whole genome sequencing on three strains (N9-2-1, N11-KP-1, and S27-4-1), each from a different individual, for pangenome analysis (Additional file 1: Table S9). Our aim was twofold: first, to compare our isolated, starvation-resistant *K. pneumoniae* to previously sequenced clinically relevant isolates, and second, to determine whether these strains encoded genomic characteristics that might account for their survival. From genome-based taxonomy, we first noticed that one of our strains designated as *K. pneumoniae* by 16S rRNA gene sequencing, strain N11-KP-1 (Fig. 3G), was in fact *K. variicola*, a closely related species to *K. pneumoniae* that can be pathogenic [21]*.* We next investigated whether isolates from healthy saliva or nares samples were phylogenetically distinct from disease-associated *K. pneumoniae,* as would be expected if these new isolates represented a novel subgroup within the *K. pneumoniae* complex. To this end, we constructed a phylogenomic bacterial marker gene tree using publicly available *K. pneumoniae* genomes from a diverse array of environments (Fig. 5A, Additional file 2: Fig. S4). While the phylogenetic tree initially contained all available 2079 complete *K. pneumoniae* and *K. variicola* genomes from NCBI RefSeq (Additional file 2: Fig. S4), it was immediately clear that these newly obtained oral and nasal isolates fell within existing clades. Guided by this overarching phylogeny, we selected a subset of reference genomes with detailed metadata for more focused analyses. This analysis indicated that our saliva and nares strains from healthy donors were most closely related to strains isolated from infected patients and hospital environments (Fig. 5A). Further, strains isolated from oral and nasal sites were intermixed with previously sequenced strains isolated from the gut, skin, and systemic infections. Thus, it does not appear that the colonization of specific body sites or pathogenicity is confined to particular clades of *K. pneumoniae.*

To better investigate these new genomes, we focused on their close relatives within the known diversity of *K. pneumoniae* and *K. variicola.* Note that these analyses do not address the population genomics of the *K. pneumoniae* complex, a topic that has been recently addressed in detail [21, 44]; instead, our analyses address whether these non-gut isolates from asymptomatic hosts share functionally similar gene content with disease-associated isolates. By constructing a pangenome to depict gene homolog presence or absence across genomes and clustering genomes based on the resultant data, the N9-2-1, N11-KP-1, and S27-4-1 isolates interspersed with other genomes from disparate isolation sources and host health statuses (Fig. 5B). Notably, N9-2-1 and S27-4-1, our two *K. pneumoniae* isolates, shared more of their accessory genomes with other clinical isolates than with each other, reflected both in the visual display and the dendrogram. N11-KP-1 was more similar in gene content to the *K. variicola* At-22 isolate from a leafcutter ant colony than to most previously described *K. variicola* included here, although the two fell within and were overall highly similar to a group of clinical isolates. All genomes, including our new genomes and the re-analyzed reference genomes, also encoded multiple antibiotic resistance pathways (Additional file 2: Fig. S5A), consistent with our antibiotic testing in Additional file 2: Fig. S2B. The tool AMRFinder predicted resistance to ß-lactams, fosfomycin, phenicol/quinolone, tetracycline, and other efflux pumps important for antibiotic resistance (Additional file 1: Table S13).

We also compared the genomes of the three isolates to a previously conducted saliva starvation experiment where *K. pneumoniae* genome mutants were tracked over 84 days [34]. Interestingly, all three of our isolates, sequenced after starvation, harbored the same nonsynonymous mutations found in Baker et al. 2019 which possibly conferred advantages to their survival (Additional file 2: Fig. S5B). Moreover, all other references *K. pneumoniae* and *K. variicola* analyzed here (Additional file 2: Fig. S5B) shared the same or similar nonsynonymous mutations, suggesting that the majority of both clinical and carriage strains share preadaptation for stress or else these mutations are selected for during routine isolation.

Overall, the *Klebsiella* isolate genomes displayed a high variability in gene content, perhaps reflecting their opportunistic lifestyle (Fig. 5B). Yet, functionally, most genomes encode similar content, including similarities in nutrient and metal uptake, and antibiotic resistance pathways (Additional file 2: Fig. S6A). This observation is supported by previous reports of *Klebsiella* species having large and highly dynamic genomes with a rich mobilome including plasmids, integrative conjugative elements, and other mobile elements [21]. Indeed, our assemblies found plasmids and mobile elements encoding genes for iron uptake (siderophores), antibiotic resistance, and more genes potentially useful to outlast and survive in harsh conditions (Additional file 1: Table S14)*.*

*Klebsiella* species are among the few oral and nasal bacteria that have a large genome size and genomic inventory, which gives them the versatility to grow on different nutrition sources and in many environments [34, 45, 46]. Based on this, we were curious if genome size could also explain why other species were also able to increase occasionally under starvation conditions (Fig. 2). To this end, we classified nares species present in our samples into two groups: (1) day-30 starvation-enriched—those that were able to achieve a relative abundance greater than 50% in any sample (Fig. 2A, D) and (2) day-30 starvation-depleted—those whose raw community mean relative abundance decreased from greater than 1% to less than 0.1% mean relative abundance within the day-30 communities. Indeed, four of the eight day-30 starvation-enriched species had a larger genome size than any raw-enriched species: *K. pneumoniae*, *K. aerogenes*, *B. anthracis*, and *C. sakazakii* (Additional file 2: Fig. S6B). Four post-starvation-enriched species, however, contained a genome with a similar size to the raw-enriched species: *K. rhizophila*, *S. warneri*, *C. pseudodiphtheriticum*, and *G. adiacens.* This may indicate that genome size does not dictate the ability to survive starvation for these species, rather that a specific gene or pathway is important.

We next performed functional enrichment analysis using whole genomes from eHOMD to assess if starvation-enriched species encoded similar metabolic pathways. Such a functional approach offers a means to disentangle genome size and functional potential, as genome size is also correlated with phylogeny and the majority of starvation-enriched isolates were Enterobacteriaceae species. This analysis pointed towards several metabolic pathways that may be correlated with starvation survivability, many of which are involved in biosynthesis (Fig. 5C). It is worth noting, however, that further experimental characterization is needed to definitively determine if these pathways are involved in starvation survivability. These genomes uniquely encoded several essential and non-essential amino acid biosynthesis and salvage pathways, including methionine, tyrosine, phenylalanine, arginine, histidine, and thiamine, as well as carbohydrate metabolism functions, including d-galactonate degradation, succinate dehydrogenase, glyoxylate cycle, and pentose phosphate cycle (Fig. 5C).

## Discussion

Exploring the ecology and adaptations of starvation-resistant bacteria may provide insight into the transmission mechanism of nosocomial pathogens since starvation conditions mimic the conditions endured within an expelled oral or nasal droplet residing on a hospital surface. Numerous studies have shown that *Klebsiella*, and other opportunistic pathogens are hardy bacteria that can survive for long periods of time on different environmental surfaces [29, 30], including hospital surfaces, sink drains, and ventilation systems [19, 20, 47, 48]. The present study suggested that the oral and nasal cavities may act as a reservoir for colonizing opportunistic pathogens such as *Klebsiella* species and examined the ability of *K. pneumoniae* to enrich in bacterial communities under starvation conditions. We demonstrated that *Klebsiella* species were exceptional and consistent in their ability to outgrow and outlast other members of the oral and nasal microbiomes under starvation conditions. Furthermore, nasal and oral *K. pneumoniae* isolates from healthy hosts were closely related to clinical strains. They shared the majority of their gene clusters, including multidrug resistance genes, pathogenicity genes, and mobile elements, with strains isolated from nosocomial infections. Compared to species that were unable to survive starvation conditions, *K. pneumoniae* and other starvation-enriched species shared several metabolic pathways involved in biosynthesis that were not encoded among bacteria unable to survive starvation conditions. Interestingly, in the absence of *Klebsiella* species, we saw oral *Peptostreptoccocus* consistently increase in relative abundance upon starvation. Much like *Klebsiella*, previous literature has implicated *Peptostreptococcus* as a pathogen involved in hospital-acquired infections and periodontal disease [42, 49, 50].

Our isolated *K. pneumoniae* strains possessed numerous antibiotic resistance genes similar to clinical isolates. These strains also demonstrated natural resistance to more than three classes of antibiotics, satisfying the criteria for multidrug resistance (MDR) [51]. In comparison, the surrounding oral and nasal microbial community members, hundreds of species, were largely susceptible to the same antibiotics and antibiotic cocktails. In our study, we harnessed a distinctive resistance profile to selectively monitor the growth of *K. pneumoniae* in mixed communities. The natural occurrence of MDR strains within these polymicrobial communities presents a potential array of risks. As suggested, multiple opportunistic pathogens that survived in our starvation experiments also have similar MDR phenotypes. These strains have the capacity to transfer their antibiotic resistance genes horizontally to other oral and nasal commensals or even to other pathogens passing through the oral cavity via our food sources [52, 53]. Once they acquire antibiotic resistance in the oral and nasal cavities, these commensals, potential pathogens, or the MDR *K. pneumoniae* strains themselves, have multiple avenues to access vulnerable infection sites, including surface contamination or saliva swallowing. Consequently, antibiotic treatments may inadvertently amplify these organisms in the microbiome or infection, intensifying the level of risk [54, 55]. Therefore, it is imperative to document and be cognizant of the presence of potentially threatening MDR oral commensal organisms.

How stable *Klebsiella* species populations are within the oral or nasal cavity remains an open question. Given their capability to survive and thrive in external environments and under harsh conditions, as demonstrated in this and other studies [29, 30], it is not illogical to consider that even a small number of patients or hospital staff harboring *Klebsiella* species in their oral or nasal communities could inadvertently become key sources of *Klebsiella* contamination across broad settings. This phenomenon could be accentuated by *Klebsiella*’s ability to thrive under starvation conditions in the presence of other members of the oral and nasal microbiomes. Our study suggests that this enrichment is attributed to *Klebsiella* species’ capacity to initially outgrow then outlive other community members. This is affirmed by *K. pneumoniae*’s ability to grow on nutrients derived from dead microbial communities and our observation that after 120 days of starvation, a substantial number of *K. pneumoniae* remained viable. Future studies may expand on our current findings by testing additional *Klebsiella* strains and species, including isolates from prestarvation conditions that have not been selected by starvation.

Supporting the possibility that oral and nasal *Klebsiella* species can disseminate, the whole genome comparison showed that the strains isolated in this study are similar to other environmental and clinic isolates. While the *K. pneumoniae* species complex is highly diverse and the genomes compared here corroborate that observation [21], the nasal and oral strains in this study do share virulence factors such as antibiotic resistance. Moreover, we do not see taxonomic clustering of *K. pneumoniae* strains from particular body sites or infection, and there is no study to date suggesting that oral and nasal *K. pneumoniae* are different from gut *K. pneumoniae*. Given the drastic differences in oxygen, nutrient availability, and microbial composition between the gut, oral, and nasal environments, the apparent lack of concomitant genetic differentiation stands in contrast to some other examples of microbial species [56–58]. With the current ability to generate MAGs from different microbiome communities and the hospital environment, future studies could track and compare oral and nasal *K. pneumoniae* directly to strains from these environmental niches.

A better understanding of why the oral and nasal cavities typically exhibit a low prevalence and abundance of *Klebsiella* species may elucidate novel ways to combat *Klebsiella* nosocomial infections. Previous analyses of the human microbiome project dataset showed that ~9% of nasal and ~3% of saliva samples contain *K. pneumoniae* [13]. Their abundance in these samples varies but is typically <1%. Our sampling of healthy oral and nasal cavities exhibited similar detection frequencies, and 6/30 (20%) nares communities and 1/30 (3.3%) saliva samples were positive for *Klebsiella* species. *Klebsiella* species colonization of human body sites can depend on multiple factors such as the ability to bind epithelial cells, evasion of immune cells, competition for resources and space with native bacteria, and direct antagonism by native bacteria. In our study, in the absence of the eukaryotic hosts and in nutrient-rich conditions, *K. pneumoniae* was unable to outgrow native community members to the same extent as the starvation condition. Further, saliva communities, which harbored much more microbial diversity than nares communities, resisted *K. pneumoniae* outgrowth during starvation better than nares communities. These results suggest that there may be commensal saliva bacteria that modulate *Klebsiella* species growth, either via nutrient competition or direct antagonism [59]. Consequently, there is a growing imperative for additional research on the ecology of *Klebsiella* and its communities. Such research holds the potential to offer insights into controlling *Klebsiella* and possibly other similar colonizing opportunistic pathogens.

## Conclusions

This study illustrates that *Klebsiella* species have the potential to enrich within the oral or nasal microbiome during starvation, and this may give an advantage outside the human oral and nasal cavity. There are numerous hospital environments that may be exposed to oral and nasal microbial communities that would result in long-term starvation, including exposed surfaces, sink drains, and mechanical ventilators, giving opportunity to *Klebsiella* for enrichment and survival. Future studies will need to address how exactly *Klebsiella* grow and maintain in a starving microbiome community and whether this contributes to the dissemination of these organisms to other body and infection sites.

## Methods

### Clinical sampling

We collected 1.5 mL of unstimulated saliva samples (named S1–S30) and anterior nares swabs (named N1–N30) from a group of 30 adults comprised of 18 females and 12 males (age range: 22–63), all of whom had well-documented medical, dental, and periodontal health records. Human subjects were recruited from subject cohorts at the Center for Clinical and Translational Research at the ADA Forsyth Institute. The study group represented the demographics of the Greater Boston and Cambridge areas with similar demographic distributions (Additional file 1: Table S15). The majority of the subjects were White (60%) and non-hispanic (90%), while 23.3% and 13.3% were Asian and Black, respectively. We excluded participants who had received periodontal disease treatment within the past 3 months, antibiotic treatment within the last 8 weeks, or antimicrobial mouthwash treatment within the last 4 weeks. Additionally, individuals with fewer than ten teeth or those currently using dentures or tobacco products were excluded from the study. The study received approval from the Forsyth Institutional Review Board under protocol number #18-06, and all subjects willingly provided written informed consent, demonstrating their voluntary participation in the research. To collect samples, unstimulated saliva was passively drooled into 15 mL conical tubes for collection, while moisten rayon-tipped swabs (Fisher Scientific #22-029-570) were inserted 1 cm into the anterior nares (separate for each nostril) and slowly rolled five times. Both swabs from the same individual were inserted into 2-mL sterile buffer saline and mixed vigorously to collect the microbiome.

### Polymicrobial community starvation

An aliquot of raw collected saliva and nares samples from each volunteer was reserved for genomic DNA isolation. The remaining fresh, non-frozen, saliva, and nares samples were grown 24 h in SHI-medium [35] in microaerophilic conditions (2% O_2_, 5% CO_2_, 93% N_2_) at 37 °C. A portion of the SHI-medium culture was reserved for genomic DNA isolation. The remaining culture was centrifuged at 18,000 x g for 10 min, and the pellet was resuspended in sterile phosphate buffer saline (PBS). Bacterial suspension in PBS derived from all 30 oral and 30 nares samples was further incubated at 37°C for 30 and 120 days with room air gas conditions with tubes sealed shut (hereafter referred to as the day-30 and day-120 communities). Following long-term starvation, a portion of these starved samples were reserved for genomic DNA isolation. The remaining samples were grown overnight in fresh SHI-medium at microaerophilic conditions at 37°C. The recovered bacteria were plated on blood agar media, and colonies were isolated and identified via Sanger 16S rRNA gene sequencing (Additional file 1: Table S9). All day-30 starvation communities were sequenced. Both day-30 and day-120 starvation communities were plated for bacterial isolation in microaerophilic gas environment.

### Genomic DNA isolation and Illumina sequencing

We isolated genomic DNA from saliva and nares samples at three time points: samples freshly collected from healthy subjects (raw communities), *in vitro* communities after expansion in SHI-medium prior to starvation (SHI-medium communities), and communities that were starved in PBS for 30 days (day-30 communities). We did not sequence day-120 communities because our previous study showed that the day-30 time point was adequate for showing the majority of the enriched species [28]. However, both day-30 and day-120 communities were grown on agar plate to determine viable bacteria and isolate enriched species (see above). Genomic DNA was isolated using the MasterPure Gram-Positive DNA purification Kit (Biosearch Technologies) with a modified protocol that included a bead-beating step [60]. Generally, Gram-positive bacteria are harder to lyse, and thus, the modified kit was used to isolate both Gram-negative and Gram-positive bacterial genomic DNA. Briefly, cells were lysed using a lysis buffer and a cell disruption machine with beads. Proteinase K and a protein precipitation reagent were added to digest and allow for the removal of protein from the sample. Genomic DNA was precipitated in isopropanol and rinsed in ethanol. Extracted DNA was stored at −80 ℃ until sequenced. Extracted DNA was sequenced by the Zymogen sequencing core on the Miseq Illumina sequencing platform targeting the V3-V4 region of the 16S rRNA gene.

### 16S RNA population profiling and analysis

16S rRNA ASVs were generated by a Zymogen in-house workflow that utilizes DADA2 [61]. ASV counts were normalized across samples using the Metacoder function “calc_obs_props” [62]. Using Metacoder, Shannon, Simpson, and Inverse Simpson alpha diversity metrics were calculated for each sample. PCoA beta diversity plots were generated using the Metacoder command “ordinate” and the specific flags “method = "PCoA", distance = "bray"”.

To assign taxonomy to the ASVs, we generated a custom Decipher classifier [63] using the most recent version of the extended Human Oral Microbiome Database (eHOMD) [41]: “https://www.google.com/url?q=https://www.homd.org/ftp/16S_rRNA_refseq/HOMD_16S_rRNA_RefSeq/V15.23/HOMD_16S_rRNA_RefSeq_V15.23.p9.fasta&sa=D&source=docs&ust=1693239952469670&usg=AOvVaw0M5dl1Ifonaz0Slth7IZWU”. To generate this custom classifier, the max group size was set to 10, and the max number of iterations were set to 3. We then used the Decipher IDTAXA approach [64] with the custom eHOMD database to assign taxonomy to the ASVs (Additional file 1: Tables S1, S2, S3, S6, S7, and S8). Taxon-level bar charts were generated using Phyloseq [65].

To compare the community member differences between the raw samples and starved samples, we generated differential heat trees using the Metacoder “heat_tree” function (Additional file 1: Table S4 and S5). Only differences with a Wilcox *p* value less than 0.05 were displayed on the trees (“false discovery rate”). To display the trees, we used the “davidson-harel” layout algorithm.

### Genomic comparisons between nares bacterial species enriched and depleted under starvation conditions

We divided the species present within our nares communities into one of three groups: (1) starvation enriched—species that achieved 50% or greater relative abundance in any starved community; (2) starvation depleted—species with a mean relative abundance of 1% or greater in raw communities and a mean relative abundance of 0.1% or less in starved communities; and (3) neutral—species that did not match the criteria for groups 1 or 2.

To assess if there was a correlation between genome size and starvation-enriched bacterial species, we used the known genome sizes listed on eHOMD. To compare the genomic inventories of starvation-enriched bacterial species to starvation-depleted bacterial species, we downloaded whole genomes of these bacterial species, when available, from eHOMD. Using anvi’o [66], we used the “anvi-estimate-metabolism” function, which employs the KEGG module database [67], to annotate each genome. We then used the anvi’o anvi-compute-metabolic-enrichment function at the default completeness threshold of 75% to identify KEGG modules associated with starvation-enriched genomes.

### Antibiotic resistance testing

Utilizing a broad array of antimicrobial compounds, we assess the antibiotic resistance of three nares polymicrobial communities from samples N3, N5, and N6; three saliva polymicrobial communities from samples S2, S5, and S8; and the three isolated strains of *Klebsiella* (N9-2-1, N11-KP-1, and S27-4-1). These were all SHI-medium communities before starvation. The selected nares and saliva communities did not contain any *Klebsiella* species by our 16S rRNA sequencing analysis. We also picked these three *K. pneumoniae* strains because *K. pneumoniae* is known to be more pathogenic than *K. aerogenes*, and they were cultured from three different individuals, representing both oral and nares strains. Resistance was assessed on brain heart infusion (BHI) supplemented with 5% sheep’s blood agar plates containing 250 μg/ml erythromycin, 50 μg/ml kanamycin, 35 μg/ml ciprofloxacin, 100 μg/ml ampicillin, 100 μg/ml streptomycin, 50 μg/ml rifampicin, 25 μg/ml nalidixic acid, 50 μg/ml vancomycin, antibiotic cocktail of *H. pylori* selective supplement (Oxoid; SR0147E), or sterile water. *H. pylori* selective supplement contained a mix of 2.5 μg/ml vancomycin, 1.25 μg/ml trimethoprim, 1.25 μg/ml cefsulodin, and 1.24 μg/ml amphotericin. Overnight cultures of *Klebsiella* strains were grown in BHI broth aerobically at 37°C. Overnight cultures of nares and saliva communities were grown in SHI-media microaerophilically at 37°C. All cultures were serially diluted from 10^−1^ through 10^−4^, plated on each medium, and incubated overnight at 37°C microaerophilically. Growth on antibiotics was graded as either resistant (indistinguishable from growth without antibiotics), some resistance (reduced confluence), little resistance (few colonies present), or susceptible (no growth observed).

### *Klebsiella pneumoniae* polymicrobial community spike-in experiment

To experimentally test the enrichment of *Klebsiella* strains during starvation, three representative nares communities (N3, N5, and N6) and three saliva communities (S2, S5, and S8) that were completely susceptible to *H. pylori* selective supplement were spiked with *Klebsiella pneumoniae* N9-2-1 and incubated in nutrient-poor PBS (phosphate buffered saline). Out of the three antibiotic tested *K. pneumoniae* strains, we used *K. pneumoniae* N9-2-1 strain specifically in our experiment since this was the only strain cultured from day-120 starvation. We reasoned that those strains would have more selective advantage, and phenotype would be clearer to observe. An overnight culture of *K. pneumoniae* N9-2-1 was grown in BHI broth microaerophilically at 37°C. *K. pneumoniae* N9-2-1 was rinsed in PBS to remove nutritional carry-over before spike-in. Overnight cultures of nares and saliva communities were grown in SHI-medium microaerophilically at 37°C. Nares and saliva community cultures were normalized to an OD600 of 1.2 (approximately equivalent to 1.9 x 10^6^ CFU/ml according to [35]), washed to remove residual growth medium, and resuspended in PBS. *K. pneumoniae* N9-2-1 was added to each community culture at approximately 0.1% (~2000 CFU/ml). This amount was estimated from our 16S rRNA sequencing analysis, which showed 0.1% to be the lower range of *Klebsiella* abundance in the community. Each *Klebsiella* spiked community was then aliquoted into 10 identical samples and incubated aerobically at 37°C. At 0 h, 8 h, 16 h, 30 h, 3 days, 12 days, 30 days, 60 days, and 120 days after initiation, one set of samples were taken out of incubation, serially diluted through 10^−8^, and plated on both BHI supplemented with 5% sheep’s blood and BHI supplemented with 5% sheep’s blood and an *H. pylori* selective supplement (Oxoid; SR0147E). Growth in the absence of antibiotic selection indicated total culturable bacteria within the community, while growth in the presence of antibiotics indicated *Klebsiella*’s contribution to the culturable community.

For 16S rRNA gene sequencing studies, we used the same protocol as our antibiotic plating assay, except on day 30, we isolated genomic DNA and sent it for sequencing at the Zymo sequencing core (Additional file 1: Table S10, S11, and S12). For the nutrition-rich passaging experiment, instead of resuspending cultures in PBS, we used SHI-medium. Cultures were grown in 96-well plates at 250 μL of final volume. Every 24 h, 50 μL of the incubated culture was passaged into a new 96-well plate containing 200 μL of fresh SHI-medium. After 30 days of passaging in a microaerophilic chamber, the culture was pelleted, and genomic DNA was sent for 16S rRNA gene sequencing.

### Whole genome sequencing and assembly

Out of the five nares and one saliva samples that had enriched *Klebsiella* after starvation, we were able to isolate two different *Klebsiella* strains from two individual nares samples (N9-2-1 and N11-KP-1) and one saliva sample (S27-4-1) (Additional file 1: Table S9). Their genomic DNA was isolated (see above gDNA isolation method) and sent for whole genome sequencing at SeqCenter following their preparation recommendations. Based on the bacterial marker gene phylogeny (see next section), one of the *K. pneumoniae* strains (N11-KP-1) was identified as *K. variicola*.

All command line parameters used during genome assembly, binning, and analysis are described in full detail and can be found with our raw data on Zenodo (see the “Data Availability” section). Briefly, each sample’s fastq files were quality trimmed with bbduk (https://sourceforge.net/projects/bbmap/) to remove adapters and drop low-quality reads before quality filtering based on the recommendations of Minoche et al. [68], using illumina-utils [69]. Genomes were assembled using SPAdes [70]. Short reads were mapped back to each assembly and incorporated into anvi’o for manual binning [71]. Contigs were binned based on tetranucleotide frequency and evenness of coverage to remove any assemblies derived from contaminant DNA from the extraction and library preparation processes. Plasmid contigs identified as extrachromosomal at the time of sequencing (based on coverage disparity) were not included in the genome bin; other mobile elements with coverage patterns consistent with genomic integration were retained. plasmidSPAdes was also used to generate assemblies of any plasmids (https://academic.oup.com/bioinformatics/article/32/22/3380/2525610).

### Comparative genomic analysis

We used an iterative phylogenetic approach to identify a small set of genomes that adequately sampled the known diversity of *Klebsiella* relative to our new isolates. Initially, a tree was constructed with GToTree [72] spanning all *K. pneumoniae* and *K. variicola* genomes on NCBI RefSeq at assembly levels of “complete” or “chromosome” and had sufficient completion to allow for successful phylogenetic reconstruction (*n*=2079 genomes) (Additional file 2: Fig. S4). Subsequently, informed by this overall phylogenetic structure, we obtained a focused set of reference genomes closely related to our new isolates that also had reliable metadata. To accomplish this, we identified all *K. pneumoniae* genomes with readily accessible metadata on NCBI to create phylogenomic trees with GToTree [72]. Oversampled clades were pruned or dropped based on the relative distance to isolate genomes, and the analysis was repeated until the genomes currently displayed were obtained. While this pruning process does remove information and could alter the overall topology of the tree, information about body site or health association, if an evolutionarily conserved trait, should persevere. *K. variicola* genomes were obtained from a recent multi-hospital analysis [73] with detailed metadata on the isolation site and host health status. *K. variicola* At-22, the leafcutter ant symbiont, was also included to represent a known environmental genome (i.e., lineages not known to associate with mammals). Two *Klebsiella* genomes were included from the Baker et al. saliva starvation experiments (84-day saliva and PBS-starved isolates) [34]. The final phylogenomic tree was generated with IQ-TREE2 [74] using the marker genes identified with GToTree.

All publicly available genomes were downloaded with “ncbi-genome-download” and incorporated into anvi’o including annotation with KEGG, Pfam, and NCBI COG [75–77]. A pangenome was constructed using “anvi-pan-genome” with “--mcl-inflation 10,” which uses the Markov clustering algorithm to cluster amino acid alignments into “gene clusters,” putatively homologous groups of genes. The presence/absence of these gene clusters was then plotted for each genome and used to compare gene content similarity via a dendrogram. Average nucleotide identity (ANI) was also computed using pyANI [78] with the blast method, and the ANI values shown reflect the percentage over the aligned regions. The presence of metabolic modules, including predicted antibiotic resistance pathways, was estimated using anvi-estimate-metabolism; based on KEGG pathways [79]. Antibiotic resistance was estimated with AMRFinderPlus using the *K. pneumoniae* model (Additional file 1: Table S13 and S14) [80].

### Supplementary Information

Additional file 1: Table S1. Relative abundances of genera identified in raw communities. Table S2. Relative abundances of genera identified in SHI-Media communities. Table S3. Relative abundances of genera found in day-30 communities. Table S4. Nares raw and day-30 community composition comparative analysis. Table S5. Saliva raw and day-30 community composition comparative analysis. Table S6. Relative abundances of species identified in Raw communities. Table S7. Relative abundances of species identified in SHI-Media communities. Table S8. Relative abundances of species found in day-30 communities. Table S9. Summary list of all isolated and identified bacteria from starvation at day 30. Identified by amplified short 16S sequence. Table S10. Relative abundances of genera identified in SHI-Media communities spiked with *Klebsiella*. Table S11. Relative abundances of genera identified in day-30 starved SHI-Media communities spiked with *Klebsiella*. Table S12. Relative abundances of genera identified in Day-30 Nutrition Rich SHI-Media communities spiked with *Klebsiella*. Table S13. AMR Finder results from *Klebsiella* isolates and previously sequenced *Klebsiella* genomes. Table S14. All functions (gene clusters) used to generate pangenome and dendrogram (Figure 5B). Table S15. Demographic characteristics of Study Population.

Additional file 2: Figure S1. Extended starvation results in homogeneous colonies. (A) Saliva directly plated on blood BHI agar shows many colony sizes and types. (B-C) After day-30 and day-120 starvation, these colonies become homogeneous in size and morphology, many displaying mucoid phenotype. Numerous images were taken and only representatives are shown. Figure S2. Characterization of isolated and cultured *Klebsiella pneumoniae* strains. (A) Artificial spiking of saliva and nares communities with *K. pneumoniae* followed by 30 day starvation resulted in increased amount of *K. pneumoniae*. After starvation, gDNA of the community was isolated and processed for DGGE. Samples were ran on large gels, and the bands were send for sequencing to identified the bacteria, as well as image was taken to quantify the band size and intensity. Total band gray area was calculated using ImageJ and plotted on the right. (B) Screening of isolated *Klebsiella* strains, nares communities, and saliva communities using a range of antibiotics identifies selective agents which can be used to isolate *Klebsiella* strains from a mixed culture. The table indicates the relative growth of each culture on the indicated antibiotic selection. Dark blue indicates robust confluent growth, lighter shades of blue indicate impaired growth, white indicates no visible growth, and grey indicates a combination which was not tested. Representative growth of the isolated *Klebsiella* strains on each tested antibiotic show strain variation in resistance profile. Figure S3. Longitudinal *K. pneumoniae* spiking and starvation experiment. (A) Serial dilution of bacterial communities from the nares (N3, N5, N6) and saliva (S2, S5, S8) which have been inoculated with an oral *K. pneumoniae* (N9-2-1) strain demonstrate rapid domination by *K. pneumoniae* when incubated in nutrient poor PBS. At 0, 8, 16, and 30 hours after starvation bacterial communities were serially diluted from 10-1 to 10-7 and 20 uL of each dilution was spotted on non-selective BHI blood plates (shown on the left) and *Klebsiella* selective media (shown on the right). *K. pneumoniae* becomes the predominant culturable bacteria within nares communities after 30 hours of starvation and within saliva communities after 8 hours. All bacterial spots were performed in technical triplicate and only representatives are shown. (B) Same experimental design as described for Figure 4F but using *K. pneumoniae *strain S27-4-1 supplemented with either 1) live (blue line), 2) heat-killed (red line) S2 saliva community, or 3) heat-killed *K. pneumoniae* strain S27-4-1(green line). Only the S27-4-1 growth on agar plate supplemented with antibiotic cocktail is shown. Mean and standard deviation are shown for 4 replicates at each time point. (C) Growth curve of S27-4-1 in BHI medium (blue) or phosphate buffer saline (PBS) (red). Data was collected for 4 replicates using a Cerillo. Figure S4. Phylogenomic tree of *Klebsiella pneumoniae* and *Klebsiella variicola* reference genomes. This tree includes the 2,079 high quality genomes from NCBI RefSeq (black text) and the three newly obtained isolates (red highlighted text). The phylogeny was constructed using an alignment of 74 core bacterial genes with an approximately maximum-likelihood algorithm (FastTree2). The tree is rooted between *K. pneumoniae* and *K. variicola*. Abbreviations: Kp: *K. pneumoniae*; Kv: *K. variicola*. Figure S5. Genome analysis of isolated *Klebsiell*a species from the starved saliva and nares samples. (A) Detection of KEGG metabolic pathways (columns) related to drug resistance, pathogenicity, and symbiosis across representative genomes (rows). Cells are colored by the proportion of each pathway’s genes present in each genome, i.e., 1 represents all genes in the pathway were detected. Genomes are arranged by isolation source and host health status (where relevant), demarcated with colored boxes on the right. (B) Amino acid residues found in each genome for the positions identified by Baker et al. 2019 as under selection during starvation. Figure S6. Global genome and genome size analysis of isolated *Klebsiella* strains. (A) Major metabolic pathways detected in representative *Klebsiella* genomes. Cells are colored by the proportion of each pathway’s genes present in each genome. Note that the figure shows only metabolic pathways with at least half (0.5) of the expected genes present. Genomes are arranged by isolation source and host health status (where relevant). (B) Genome of bacterial species enriched in raw (blue) or day-30 starved (orange) samples were obtained from the eHOMD. The size of the genomes were plotted against all other oral and nasal genomes (gray) from the eHOMD.

## Data Availability

All relative abundance data are provided in the manuscript supplemental tables. Raw nucleic acid sequences and code used in this project are available on Zenodo (DOI:10.5281/zenodo.10403630; 10.5281/zenodo.10403629) and GitHub (https://github.com/jett-liu/Persistent-enrichment-of-multidrug-resistant-Klebsiella.git). The raw data and code are also available at https://www.borlab.org/resources. Bacterial strains used in this paper will be provided upon request.

## Abbreviations

COPs: Colonizing opportunistic pathogens
ESKAPE: *Enterococcus faecium*, *Staphylococcus aureus*, *Klebsiella pneumoniae*, *Acinetobacter baumanni*, *Pseudomonas aeruginosa*, and *Enterobacter* spp.
PBS: Phosphate buffer saline
BHI: Brain heart infusion
eHOMD: expanded Human Oral Microbiome Database
CFU: Colony-forming units
