# Supplementary material for: Persistent enrichment of multidrug-resistant Klebsiella in oral and nasal communities during long-term starvation
Source: Microbiome. 2024 Jul 20;12:132. doi: 10.1186/s40168-024-01854-5 (PMC11264962; doi:10.1186/s40168-024-01854-5)
Supplement: Supplementary file 3 — Additional file 2: Figure S1. Extended starvation results in homogeneous colonies. (A) Saliva directly plated on blood BHI agar shows many colony sizes and types. (B-C) After day-30 and day-120 starvation, these colonies become homogeneous in size and morphology, many displaying mucoid phenotype. Numerous images were taken and only representatives are shown. Figure S2. Characterization of isolated and cultured Klebsiella pneumoniae strains. (A) Artificial spiking of saliva and nares communities with K. pneumoniae followed by 30 day starvation resulted in increased amount of K. pneumoniae. After starvation, gDNA of the community was isolated and processed for DGGE. Samples were ran on large gels, and the bands were send for sequencing to identified the bacteria, as well as image was taken to quantify the band size and intensity. Total band gray area was calculated using ImageJ and plotted on the right. (B) Screening of isolated Klebsiella strains, nares communities, and saliva communities using a range of antibiotics identifies selective agents which can be used to isolate Klebsiella strains from a mixed culture. The table indicates the relative growth of each culture on the indicated antibiotic selection. Dark blue indicates robust confluent growth, lighter shades of blue indicate impaired growth, white indicates no visible growth, and grey indicates a combination which was not tested. Representative growth of the isolated Klebsiella strains on each tested antibiotic show strain variation in resistance profile. Figure S3. Longitudinal K. pneumoniae spiking and starvation experiment. (A) Serial dilution of bacterial communities from the nares (N3, N5, N6) and saliva (S2, S5, S8) which have been inoculated with an oral K. pneumoniae (N9-2-1) strain demonstrate rapid domination by K. pneumoniae when incubated in nutrient poor PBS. At 0, 8, 16, and 30 hours after starvation bacterial communities were serially diluted from 10-1 to 10-7 and 20 uL of each dilution was spot [file 40168_2024_1854_MOESM2_ESM.docx]

**Supplemental Figures**

**
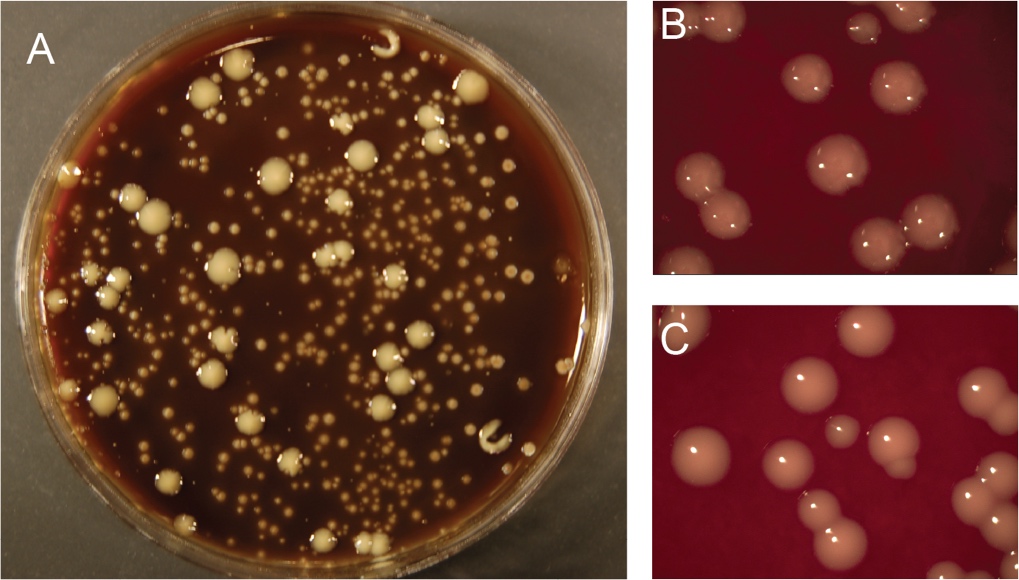
**

**Figure S1.** Extended starvation results in homogeneous colonies. (A) Saliva directly plated on blood BHI agar shows many colony sizes and types. (B-C) After day-30 and day-120 starvation, these colonies become homogeneous in size and morphology, many displaying mucoid phenotype. Numerous images were taken and only representatives are shown.

**
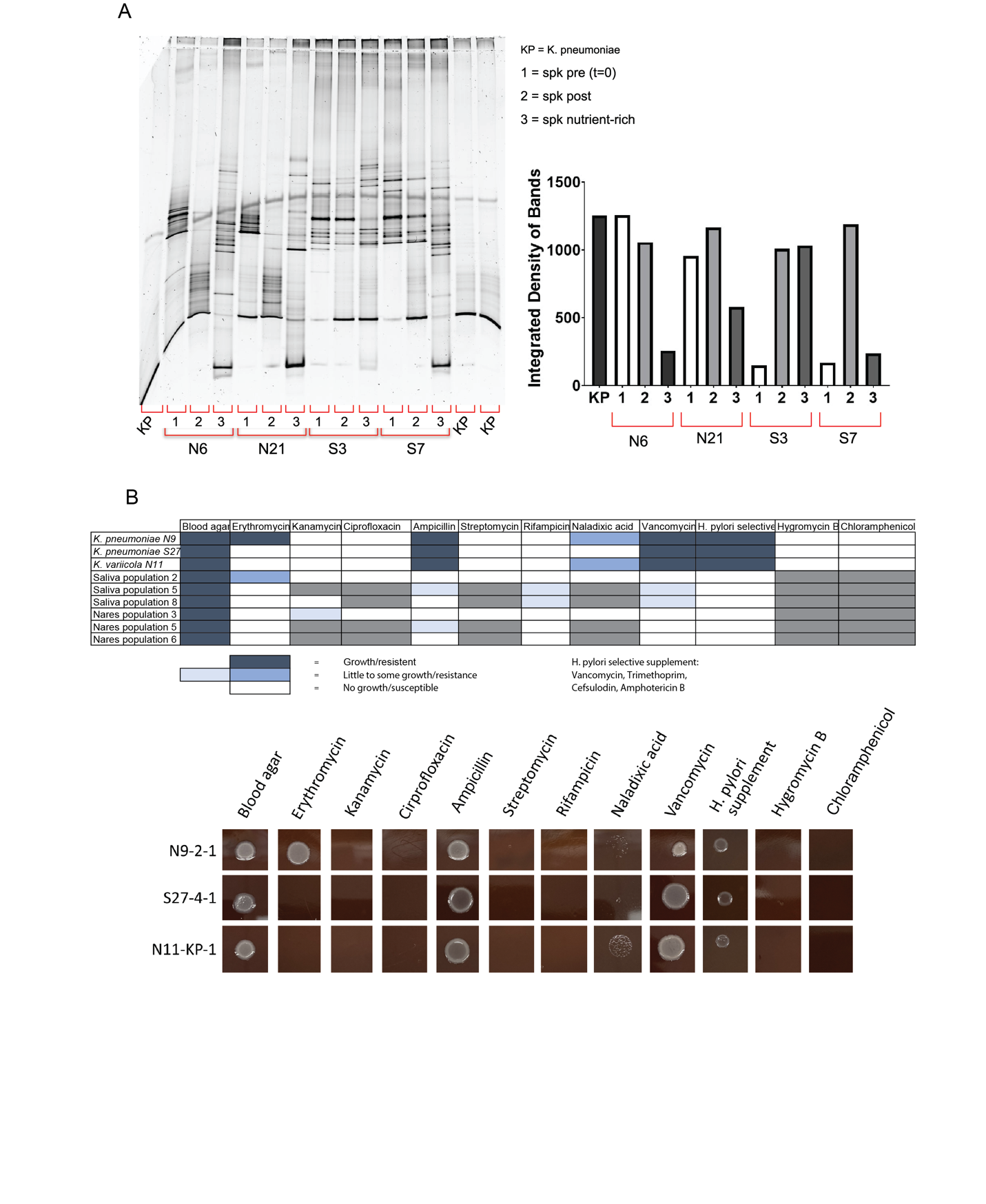
**

**Figure S2.** Characterization of isolated and cultured *Klebsiella pneumoniae* strains. (A) Artificial spiking of saliva and nares communities with *K. pneumoniae* followed by 30 day starvation resulted in increased amount of *K. pneumoniae.* After starvation, gDNA of the community was isolated and processed for DGGE. Samples were ran on large gels, and the bands were send for sequencing to identified the bacteria, as well as image was taken to quantify the band size and intensity. Total band gray area was calculated using ImageJ and plotted on the right. (B) Screening of isolated *Klebsiella* strains, nares communities, and saliva communities using a range of antibiotics identifies selective agents which can be used to isolate *Klebsiella* strains from a mixed culture. The table indicates the relative growth of each culture on the indicated antibiotic selection. Dark blue indicates robust confluent growth, lighter shades of blue indicate impaired growth, white indicates no visible growth, and grey indicates a combination which was not tested. Representative growth of the isolated *Klebsiella* strains on each tested antibiotic show strain variation in resistance profile.

**
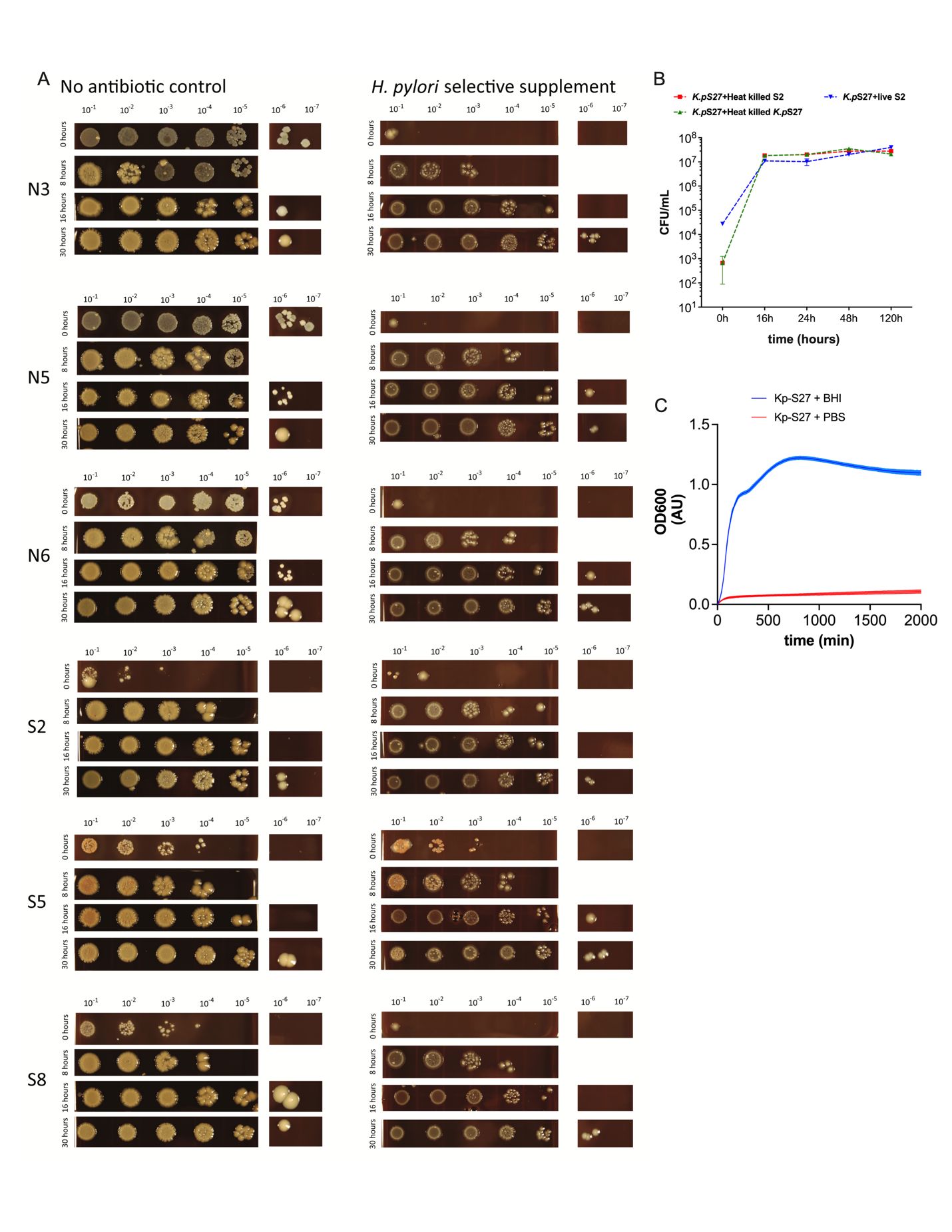
**

**Figure S3.** Longitudinal *K. pneumoniae* spiking and starvation experiment. (A) Serial dilution of bacterial communities from the nares (N3, N5, N6) and saliva (S2, S5, S8) which have been inoculated with an oral *K. pneumoniae* (N9-2-1) strain demonstrate rapid domination by *K. pneumoniae* when incubated in nutrient poor PBS. At 0, 8, 16, and 30 hours after starvation bacterial communities were serially diluted from 10^-1^ to 10^-7^ and 20 μL of each dilution was spotted on non-selective BHI blood plates (shown on the left) and *Klebsiella* selective media (shown on the right). *K. pneumoniae* becomes the predominant culturable bacteria within nares communities after 30 hours of starvation and within saliva communities after 8 hours. All bacterial spots were performed in technical triplicate and only representatives are shown. (B) Same experimental design as described for Figure 4F but using *K. pneumoniae* strain S27-4-1 supplemented with either 1) live (blue line), 2) heat-killed (red line) S2 saliva community, or 3) heat-killed *K. pneumoniae* strain S27-4-1(green line). Only the S27-4-1 growth on agar plate supplemented with antibiotic cocktail is shown. Mean and standard deviation are shown for 4 replicates at each time point. (C) Growth curve of S27-4-1 in BHI medium (blue) or phosphate buffer saline (PBS) (red). Data was collected for 4 replicates using a Cerillo.


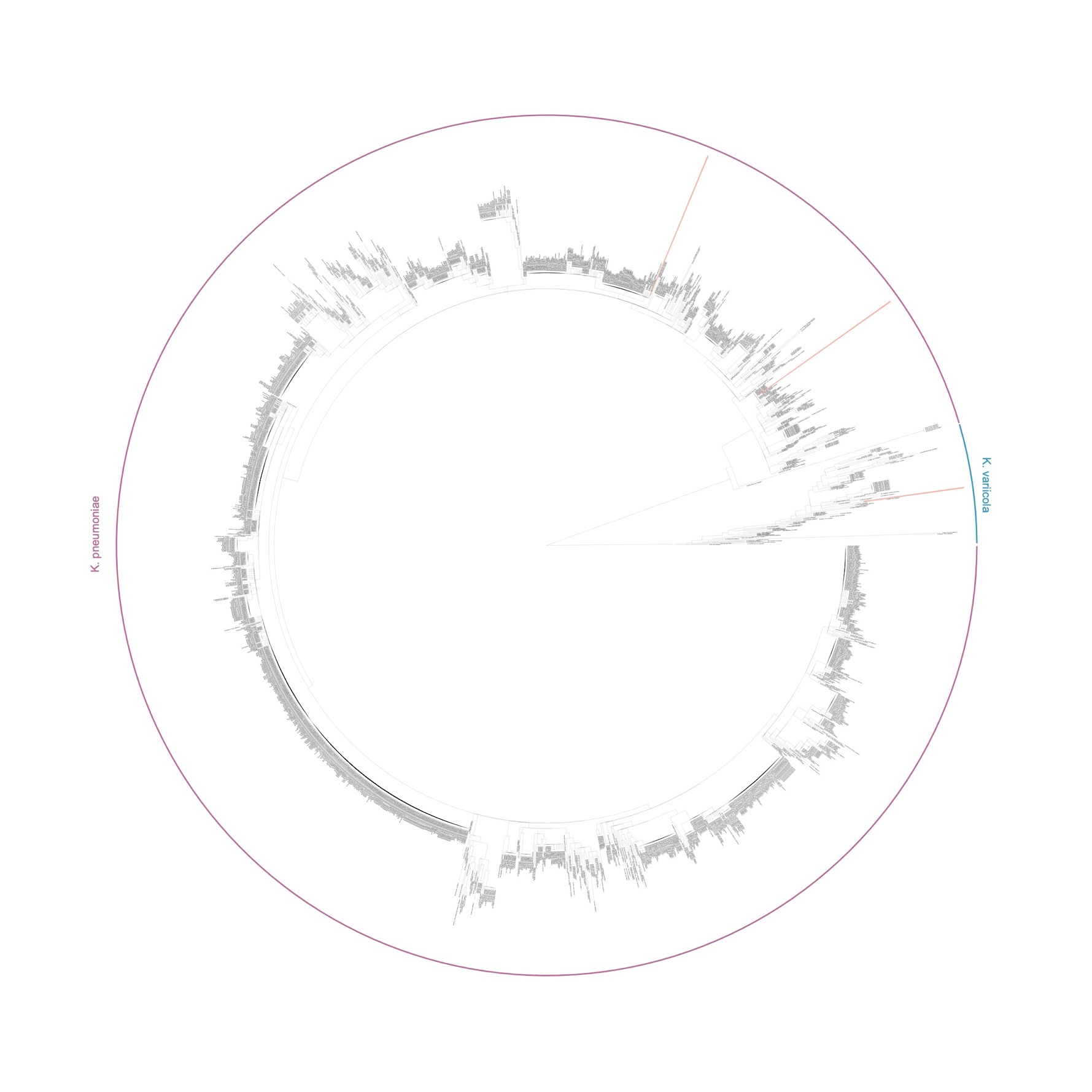


**Figure S4**. Phylogenomic tree of *Klebsiella pneumoniae* and *Klebsiella variicola* reference genomes. This tree includes the 2,079 high quality genomes from NCBI RefSeq (black text) and the three newly obtained isolates (red highlighted text). The phylogeny was constructed using an alignment of 74 core bacterial genes with an approximately maximum-likelihood algorithm (FastTree2). The tree is rooted between *K. pneumoniae* and *K. variicola*. Abbreviations: Kp: *K. pneumoniae*; Kv: *K. variicola*.

**
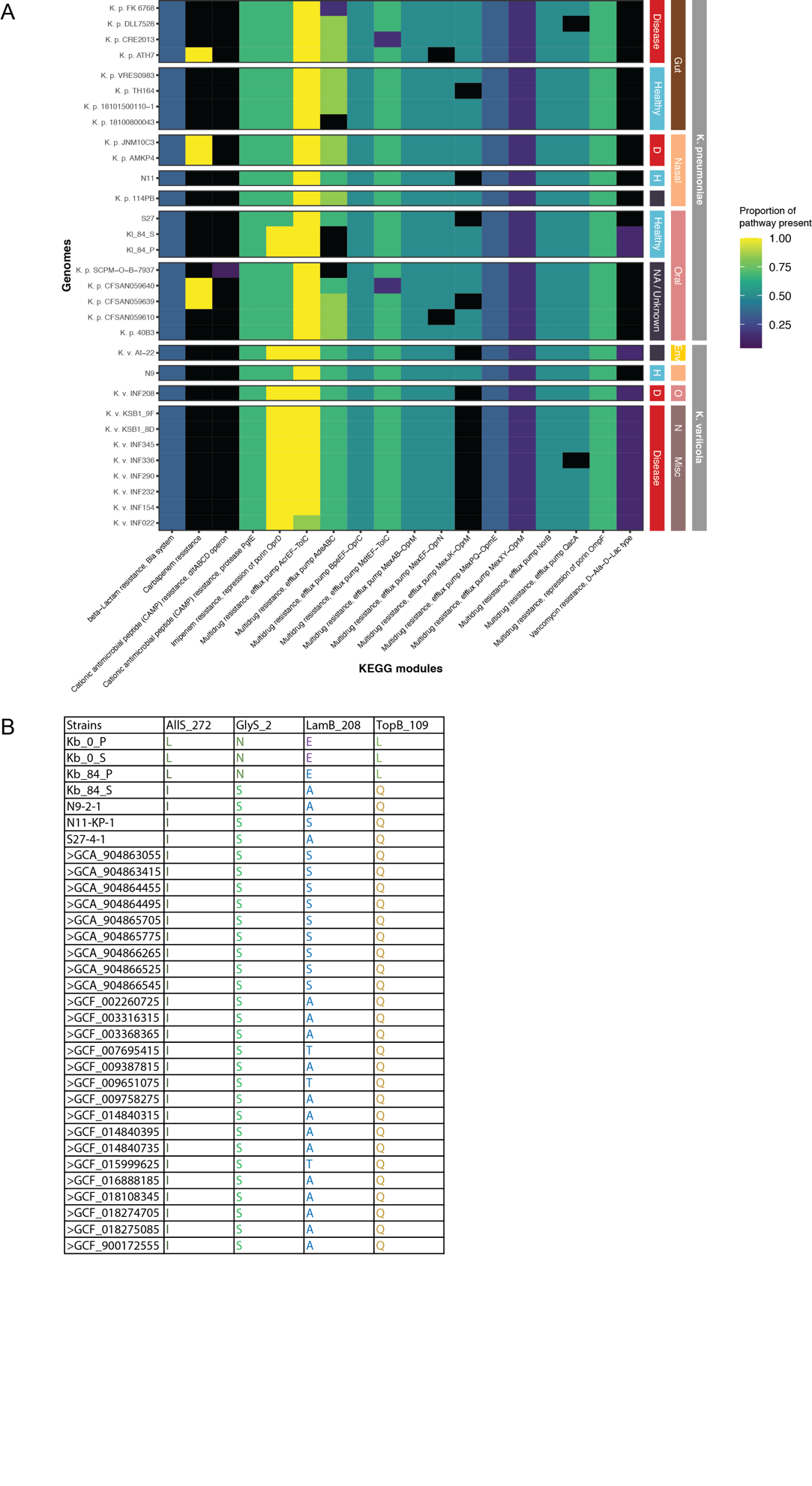
**

**Figure S5.** Genome analysis of isolated *Klebsiella* species from the starved saliva and nares samples. (A) Detection of KEGG metabolic pathways (columns) related to drug resistance, pathogenicity, and symbiosis across representative genomes (rows). Cells are colored by the proportion of each pathway’s genes present in each genome, i.e., 1 represents all genes in the pathway were detected. Genomes are arranged by isolation source and host health status (where relevant), demarcated with colored boxes on the right. (B) Amino acid residues found in each genome for the positions identified by Baker et al. 2019 as under selection during starvation.

**
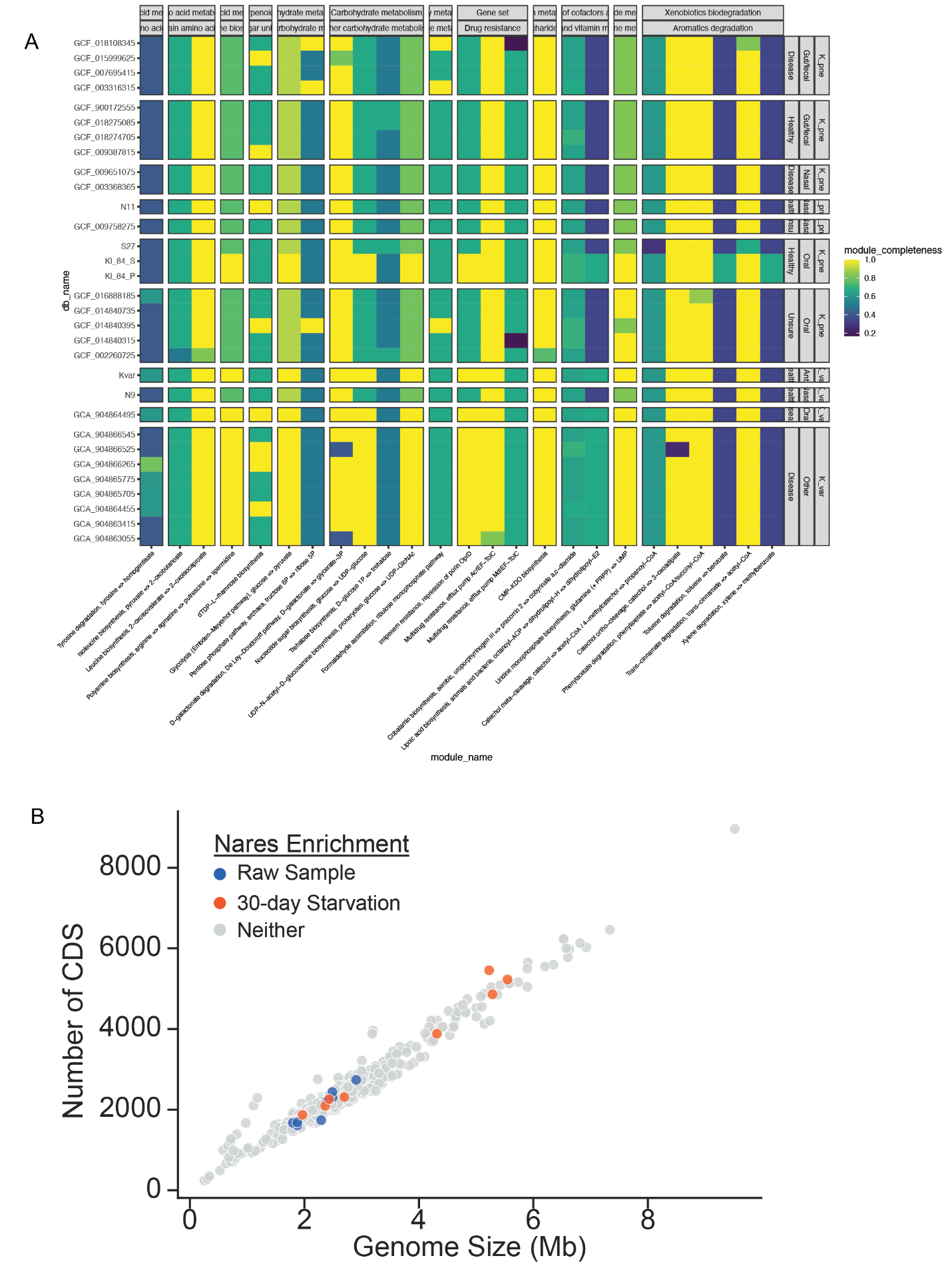
**

**Figure S6.** Global genome and genome size analysis of isolated *Klebsiella* strains. (A) Major metabolic pathways detected in representative *Klebsiella* genomes. Cells are colored by the proportion of each pathway’s genes present in each genome. Note that the figure shows only metabolic pathways with at least half (0.5) of the expected genes present. Genomes are arranged by isolation source and host health status (where relevant). (B) Genome of bacterial species enriched in raw (blue) or day-30 starved (orange) samples were obtained from the eHOMD. The size of the genomes were plotted against all other oral and nasal genomes (gray) from the eHOMD.
